# Using available *in vitro* metabolite identification and time course kinetics for β-chloroprene and its metabolite, (1-chloroethenyl) oxirane, to include reactive oxidative metabolites and glutathione depletion in a PBPK model for β-chloroprene

**DOI:** 10.3389/fphar.2023.1223808

**Published:** 2023-08-17

**Authors:** J. L. Campbell, H. J. Clewell, C. Van Landingham, P. R. Gentry, M. E. Andersen

**Affiliations:** ^1^ Ramboll US Corporation, Raleigh, NC, United States; ^2^ Ramboll US Corporation, Monroe, LA, United States; ^3^ Andersen ToxConsulting, LLC, Chapel Hill, NC, United States

**Keywords:** chloroprene, reactive metabolites, PBPK, cancer mode of action, glutathione depletion

## Abstract

**Introduction:** ß-chloroprene (2-chloro-1,3-butadiene; CP) causes lung tumors after inhalation exposures in rats and mice. Mice develop these tumors at lower exposures than rats. In rats CP exposures cause depletion of lung glutathione (GSH).

**Methods:** PBPK models developed to relate the appearance of mouse lung tumors with rates of CP metabolism to reactive metabolites or total amounts metabolized during exposures have been expanded to include production of reactive metabolites from CP. The extended PBPK model describes both the unstable oxirane metabolite, 2-CEO, and metabolism of the more stable oxirane, 1-CEO, to reactive metabolites via microsomal oxidation to a diepoxide, and linked production of these metabolites to a PK model predicting GSH depletion with increasing CP exposure. Key information required to develop the model were available from literature studies identifying: 1) microsomal metabolites of CP, and 2) *in vitro* rates of clearance of CP and 1-CEO from active microsomal preparations from mice, rats, hamsters and humans.

**Results:** Model simulation of concentration dependence of disproportionate increases in reactive metabolite concentrations as exposures increases and decreases in tissue GSH are consistent with the dose-dependence of tumor formation. At the middle bioassay concentrations with a lung tumor incidence, the predicted tissue GSH is less than 50% background. These simulations of reduction in GSH are also consistent with the gene expression results showing the most sensitive pathways are Nrf2-regulation of oxidative stress and GSH metabolism.

**Discussion:** The PBPK model is used to correlate predicted tissue exposure to reactive metabolites with toxicity and carcinogenicity of CP.

## 1 Introduction

A physiologically-based pharmacokinetic (PBPK) model was previously developed for chloroprene (CP; 2-chloro-1,3-butadiene) to estimate lung dose metrics and correlate these dose metrics with toxicity and carcinogenicity in the mouse (Clewell et al., 2019; 2020). The dose metric calculated with this PBPK model was total amount of CP metabolized per gram of lung. This dose metric considers only one aspect of tissue exposure to reaction products of CP, i.e., their production rates. More complete consideration of tissue dose should include production of the reactive metabolites, their clearance from tissue and any considerations of dose-dependence of clearance, such as limitations on the concentrations of glutathione (GSH)—a protective tissue sulfhydryl.

Inhalation exposure of rats to CP caused depletion of tissue GSH in both liver and lung (Plugge and Jaeger, 1979). PBPK modeling with other chlorinated alkenes, such as vinyl chloride and vinylidene chloride, have considered the conjugation of reactive products with GSH and the background synthesis and loss of tissue GSH (D’Souza and Andersen, 1988; D’Souza et al., 1988; Clewell et al., 2001). These models do not make predictions of the exact concentration of transient reactive products in tissues but permit simulation of GSH concentrations, proportionate exposures to reactive products and how these tissue exposures to reactive products disproportionately increase with depletion of tissue GSH. As GSH levels fall, the concentration of reactive metabolites increases disproportionately with exposure, leading to exacerbation of toxic responses in the tissue because reactive products with various chemistries then are more available to react with cellular constituents other than GSH. The expectations of increased tissue toxicity with GSH depletion are consistent with observations of liver and lung responses in fasted rats to CP inhalation (Jaeger et al., 1975; Plugge and Jaeger, 1979) and similar responses in livers in fasted rats exposed to 1,1-DCE (McKenna et al., 1977; Andersen et al., 1980).

Oxiranes produced by microsomal oxidation of ethenes, such as ethylene oxide or butadiene monoxide and butadiene diepoxide, are sufficiently stable to undergo Phase II metabolism by epoxide hydrolases (EHs) and glutathione transferases (GSTs) and to diffuse from the tissues where they are produced into the bloodstream for transport to other tissues (Johanson and Filser, 1993; Kohn and Melnick, 2000; Filser and Klein, 2018). In contrast, for halogen substituted alkenes, such as CP, vinylidene chloride and vinylidene bromide, the oxiranes formed with a halogen on one of the carbons in the oxirane are very unstable and are predicted to rapidly rearrange producing a highly reactive haloacetylhalide (D’Souza and Andersen, 1988; Forkert, 1999a; Forkert, 1999b; USEPA, 2002). Of the two reaction products formed by the microsomal oxidation of CP, (1-chloroethenyl) oxirane (1-CEO) is expected to have a short, but measurable half-life in tissues. (2-chloroethenyl) oxirane (2-CEO), on the other hand, is expected to quickly rearrange to reactive products produced by rearrangement of both 2-CEO and any diepoxide that might be produced from further oxidation of 1-CEO (Himmelstein et al., 2004).

Extending the CP PBPK model (Clewell et al., 2019; Clewell et al., 2020) to include the production of various reactive intermediates requires detailed information on rates of *in vitro* metabolism and identification of various metabolites of CP formed by microsomal metabolism (Cottrell et al., 2001; Munter et al., 2003; Himmelstein et al., 2004; Munter et al., 2007). In a study to speciate metabolites (Munter et al., 2003), the main metabolites identified were the diol produced by epoxide hydrolase mediated hydration of 1-CEO and pathways to a variety of reactive aldehydes and ketones formed from 2-CEO were noted by identification of conjugates that were found both in the presence and absence of glutathione transferase. There was no evidence for formation of a stable CP diepoxide with microsomes from any of the tested species. The elegant methods used by Munter et al. (2003) allowed determination of specific metabolites and their relationships to the initial steps of CP oxidation. One limitation was the method of incubation. Microsomes from mice, rat and human were incubated with CP (0.01—10 mM) dissolved in 5 µL acetonitrile in a 1 mL incubation volume. The mixture was then placed in a 10 mL gastight vial for 30 min at 37°C. Due to partitioning between the air and gas phase and the presence of acetonitrile, it is not possible to extract kinetic information useful for the PBPK model. The incubation concentrations are difficult to estimate and the possible kinetic interactions between CP and acetonitrile are likely to obscure more quantitative results for establishing kinetic constants. Acetonitrile is metabolized by a cytochrome P450 dependent pathway (Freeman and Hayes, 1987) and would be expected to compete with CP as a substrate for metabolism.

Other studies looking at loss of CP or appearance of 1-CEO in a headspace above a 1 mL incubation mixture in a 10 mL airtight vial, such as those from Himmelstein et al. (2004), provided more quantitative information for assessing kinetic constants for CP oxidation and 1-CEO production and clearance. Using lung microsomes, approximately 5%–20% of total oxidative metabolism produced 1-CEO. While the other 80%–95% of reaction products were not characterized, they presumably represent production of 2-CEO. The 2-CEO must react much more rapidly in the liquid phase since none was found in the headspace and only GSH conjugates of expected reactive metabolites rather than the metabolites themselves were directly measured. With mouse microsomes, but not microsomes from other species, there were two pathways for clearance of 1-CEO from the head space, water addition by epoxide hydrolase and an NADP + -dependent pathway, presumably CYP-P450 mediated oxidation of 1-CEO to a diepoxide that would then also rapidly rearrange to reactive aldehydes and ketones that react with GSH.

Relying on the results from these two papers (Munter et al., 2003; Himmelstein et al., 2004) for describing metabolism and amounts of metabolites available for reaction with GSH, and merging the CP PBPK model from Clewell et al. (2019) and PBPK models for glutathione production, turnover and reactivity with reactive products (D’Souza and Andersen, 1988; Clewell et al., 2001), allows refinement and extension of the earlier PBPK model for CP (Clewell et al., 2019; 2020) to develop model descriptions of reactive metabolite production, reaction of these metabolites with glutathione and conditions under which GSH depletion is expected to lead to disproportionate increases in adduction of reactive metabolites with tissue nucleophiles other than GSH.

The goal of this present effort was to extend the PBPK model for CP to include more detail on the epoxides and other reactive products formed by oxidative metabolism, to describe the impact of production of these reactive products on tissue glutathione (GSH) in mice and humans, and apply the integrated model to correlate predicted tissue exposure to reactive metabolites with toxicity and carcinogenicity of CP.

## 2 Materials and methods

**FIGURE 1:**
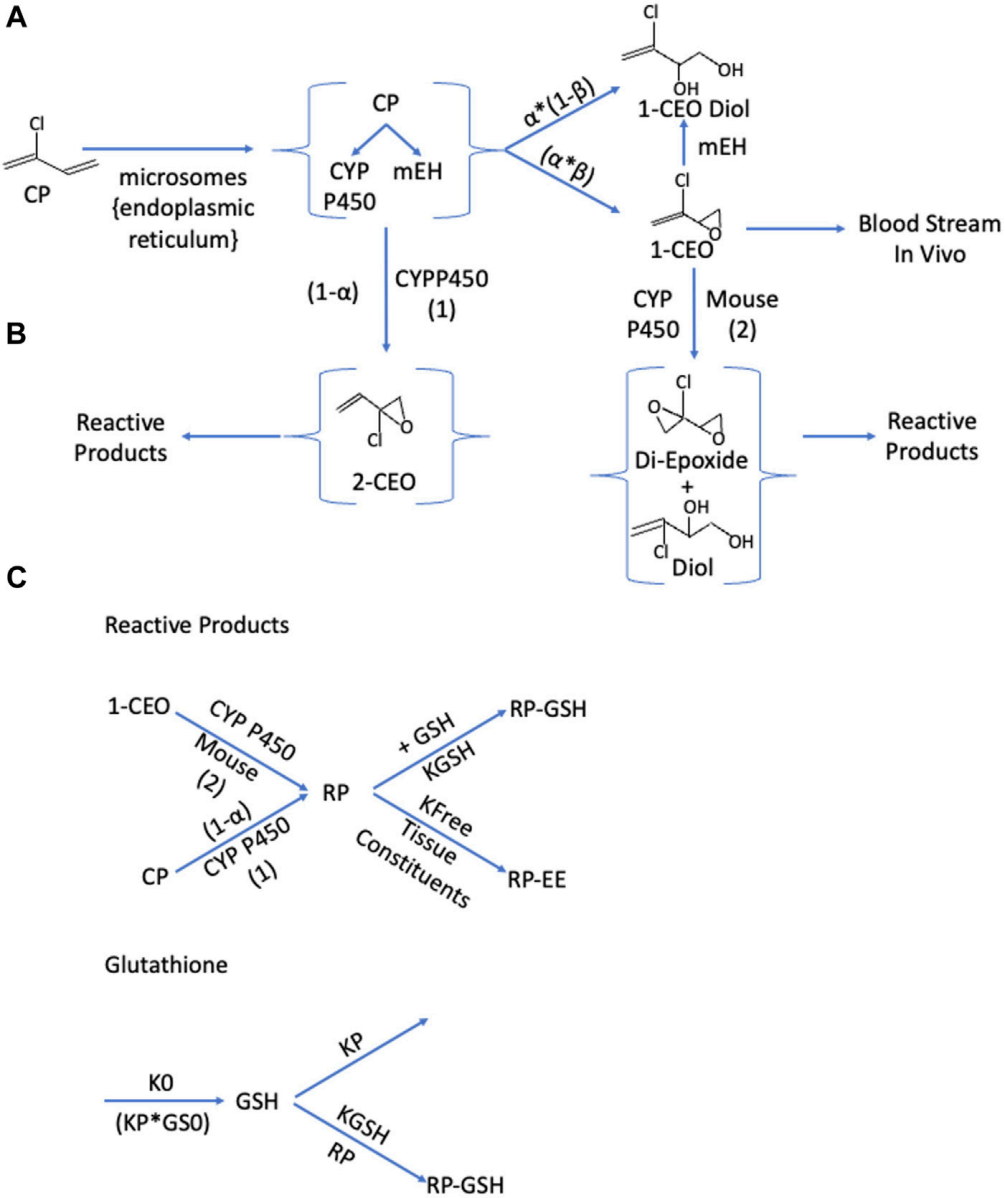
Schematics for the production and clearance of the three key components in the enhanced PBPK model for chloroprene (CP). **(A)**: Microsomal Oxidation of Chloroprene Produces Reactive Intermediates. Metabolism of chloroprene (2-chloro-1,3-butadiene) produces both 1-chloroethenyloxirane (1-CEO) and 2-chloro-2ethenyl oxirane (2-CEO). The proportion of oxidation producing 1-CEO is α; the proportion producing 2-CEO is (1-α). Microsomes contain both the cytochromes responsible for oxidation to these oxirane and microsomal epoxide hydrolase (m-EH) that can add water to the epoxide. These enzyme activities are collocated in the endoplasmic reticulum and not all the material produced by oxidation is free for release to the cytoplasm. A portion is directly available as the oxirane (β); and the remainder (1-β) is released as 1-CEO-diol. Tissue 1-CEO can be released into the bloodstream, and in the mouse, further oxidation produces a di-epoxide. **(B)**: The pathway to 2-CEO and to the diepoxide or diepoxide diol produced by a oxidation of 1-CEO, produces a variety of reactive aldehydes and ketones (Munter et al., 2007), collectively referred to as reaction products (RP). RP react with various tissue constituents with a rate constant, kfee, and with glutathione (GSH) with a second order rate constant, Kgsh. **(C)**: Cellular steady-state conditions for glutathione (GS) are maintained where a zero-order production rate (KO) equals the loss rate GSH (KP*GS0). The presence of reactive products reacting with glutathione leads to a shift to lower levels of tissue GSH.

Formation and clearance processes with 1-CEO: 1-CEO is formed by the oxidation of CP by cytochrome P450 enzymes, primarily CYP2f1 and 2e1 (Figure 1A). This oxidation step produces both 1- and 2-CEO and the relative split for the flux through both pathways was estimated separately for liver and lung microsomes (Himmelstein et al., 2004). In the subsequent equations, alpha (a) is the proportion of CYP-oxidation producing 1-CEO, and (1-a) is the proportion producing 2-CEO (Figure 1B). In addition, the products of the first oxidation step include both the respective epoxide and diol due to the proximity of the cytochrome P450s and microsomal epoxide hydrolases within the microsomes, i.e., within the endoplasmic reticulum in the intact tissues. Thus, some diol is produced by an intracellular first-pass-like process where the proximity of the CYP enzymes and epoxide hydrolase in microsomal vesicles allows some direct conversion of the epoxide to the diol before release from the lipophilic environment of the microsome to the cytoplasm (Johanson and Filser, 1993; Kohn and Melnick, 2000). An estimate of the proportion of diol produced by the oxidation (b) was available from modeling with butadiene (Campbell et al., 2015). The subsequent clearance of 1-CEO occurs by three pathways in the mouse, EH/H_2_O hydrolysis, further microsomal oxidation of 1-CEO (Himmelstein et al., 2004) and, *in vivo*, diffusion of 1-CEO from tissue into the bloodstream. While reactions of 1-CEO with GSH, either catalyzed by glutathione-S-transferases or by direct non-enzymatic conjugation, are possible, there was no evidence for this pathway in human, rat, or mouse microsomal incubations (Munter et al., 2003). Furthermore, with human microsomal preparations, there was no evidence of a second oxidation step consuming 1-CEO in time course studies of appearance or loss of 1-CEO from the closed head space above microsomal suspensions (Himmelstein et al., 2004).

Kinetic constants for CP oxidation were estimated from these *in vitro* studies following the loss of headspace CP from vials containing microsomal suspensions. Those for 1-CEO oxidation followed 1-CEO headspace loss using microsomal suspensions with added NADPH. 1-CEO hydrolysis was also assessed using microsomal preparations with no added NADPH. In these detailed kinetic studies of multiple pathways (Himmelstein et al., 2004), GSH conjugation was examined by evaluating loss of headspace 1-CEO with vials containing cytoplasm and 10 mM GSH—a GSH level about 5 times higher than background levels in lung (Jaeger et al., 1974; Csanady et al., 2003). As noted in studies identifying metabolites of CP (Munter et al., 2003), there was no evidence for appreciable clearance of 1-CEO by reactions involving glutathione.

*In vitro* derived metabolic rate constants for liver and lung were scaled allometrically using Eq. 1. Briefly, the *in vitro* constant was scaled to the protein content and tissue volume and then allometrically (BW^0.75^) for application in the CP PBPK model. The parameters used for the female mouse and rat were taken from Brown et al., (1997) and Marino et al. (2006) as reported in Supplementary Table S1.

$$\begin{array}{ll} In\;vivo\;Vmaxc\;\left(mg/h/kg\;BW^{0.75}\right) & =in\;vitro\;Vmax\;\left(\mu mol/h/mg\;microsomal\;protein\_tissue\right)\\ & x\;MPPGL\;\left(mg\;microsomal\;protein\_tissue/g\;tissue\right)\\ & x\;BW\;\left(kg\right)\;x\;VtissueC\;x\;1000\;\left(g/kg\right)÷BW^{0.75}\\ & x\;MW\;\left(\mu g/umol\right)÷1000\;\left(\mu g/mg\right) \end{array}$$ (1)

Formation and clearance processes with 2-CEO: Similar to 1-CEO, 2-CEO would be an intermediate of the oxidation of CP by CYP2e1 and 2f1 (Figure 1B). The proportion of 2-CEO formed is (1-α) times the net rate of loss of CP, where α is the proportion of the initial oxidation step that goes to 1-CEO. As with the formation of 1-CEO and 1-CEO diol, the proportion of the formation of the diol would be ß times the total oxidation rate. However, neither 2-CEO nor 2-CEO diol is sufficiently stable to remain in tissue and are expected to undergo rapid rearrangement to reactive aldehydes and ketones, all of which react with GSH (Munter et al., 2003). Modeling conjugation of reactive products with glutathione was similar to that used in PBPK models for reactive products with vinyl chloride (Clewell et al., 2001) and vinylidene chloride (D'Souza et al., 1988). In addition, the oxidative reaction of 1-CEO to a diepoxide, a pathway only present with mouse microsomes (Himmelstein et al., 2004) would also produce various reactive aldehydes and ketones that rapidly conjugate with GSH The net flux of all these reactive intermediates is captured in a single lumped compartment, called the reactive product (RP) pool.

**FIGURE 2:**
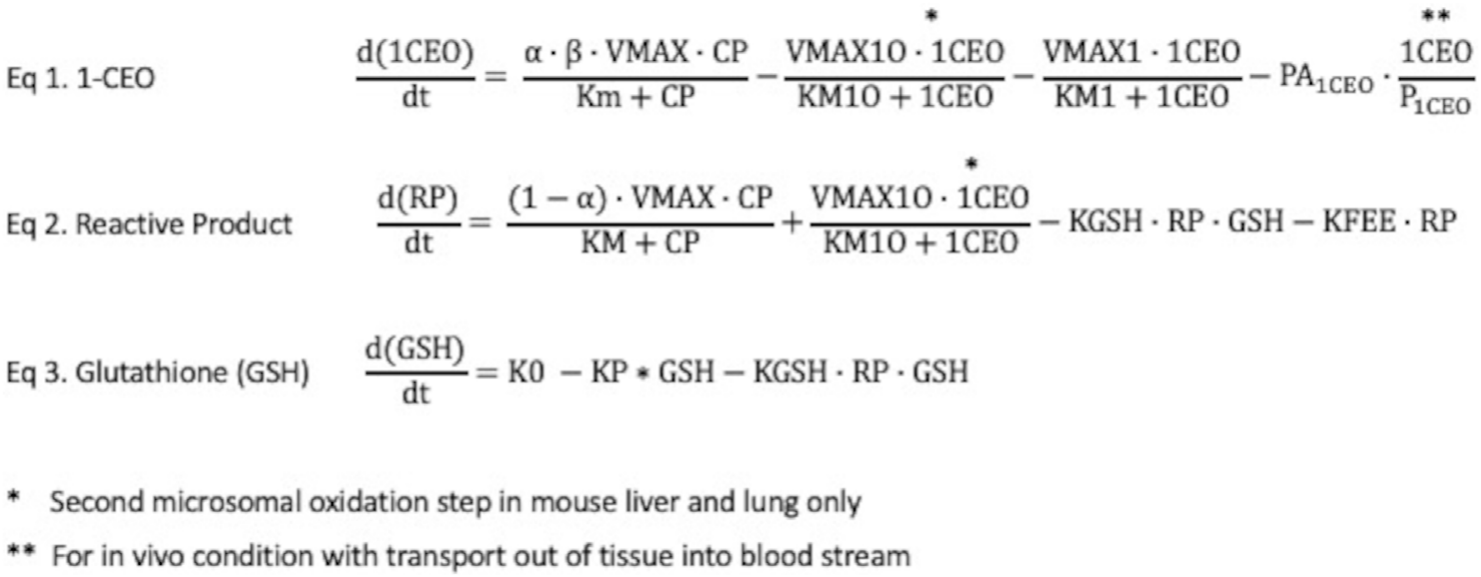
Rate equations for 1-CEO, reactive products (RPs) and glutathione (GSH) in liver and lung. Eq. 1: The rate equation for formation and clearance of 1-CEO includes 4 terms—net production by microsomal oxidation, oxidation of 1-CEO to a diepoxide, a process only present in liver, not in lung; hydrolysis by epoxide hydrolases and diffusion out of lung tissue to venous blood, where PA1_CEO_ is the permeation area cross-product for the rate of diffusion across tissue barriers into blood. This last term is only applicable to simulations in the living mouse. Eq. 2: The rate equation for production and clearance of reactive products also has 4 terms—production from the oxidation of CP, production in liver from second oxidation step for 1-CEO, loss by reactions with GSH and loss by reactions with a variety of tissue constituents. Eq. 3: The rate equation for tissue GSH includes a zero-order synthesis rate and terms for the reactions of GSH with reactive products and the basal process of cellular utilization of GSH, modeled with a first-order rate constant. The cellular steady state GSH is simply K0/KP.

Formation and tissue clearance of transient RPs: The RP pool represents a diverse group of reactive aldehydes and ketones that form by rearrangement of the unstable epoxide, 2-CEO, and of the diepoxide formed by oxidation of 1-CEO (Figures 1, 2). These products themselves are expected to be relatively short-lived and unlikely to appreciably diffuse out of the cells and into the blood, due to their rapid reaction with GSH and other cellular components. The GSH conjugation pathway is favored at normal GSH concentrations. The rate equation for RPs would have the net rates of production of 2-CEO and the 1-CEO diepoxide and loss due to reaction with GSH and with other cellular constituents. The abbreviation, KFEE, represents a first order rate constant for reaction of RPs with “everything else”. A first-order constant is used because the reaction of RPs with these other tissue components, though the driver of tissue toxicity, is not expected to deplete the total reactant pool of these constituents to any great extent. A similar approach for modeling reactive intermediates was used with vinyl chloride (Clewell et al., 2001).

Depletion of GSH: The last process that needed to be included in the model was the production and removal of RPs and the effect of higher rates of formation of RPs on GSH (Figure 1C). Higher rates of formation of RPs are expected to cause depletion of GSH leading to increased tissue toxicity from these RPs. The rate constants for GSH synthesis (Ko) and background loss (k1) have been approximated in various previous publications with vinyl chloride, ethylene dichloride and vinylidene chloride (D'Souza and Andersen, 1988; D'Souza et al., 1988) and specifically for mouse lung in publications with styrene and styrene oxide (Csanady et al., 2003). These rate constants are independent of any reactions with exogenous chemicals, which must be added to the mass balance equation for GSH. With such compounds, there is depletion of tissue GSH, usually measured in liver, following higher exposures. Depletion of non-protein sulfhydryls (NPS) in lung tissue was reported in fasted rats exposed to 100 or 300 ppm CP (Plugge and Jaeger, 1979) and the most sensitive gene ontology pathways affected by CP exposures in mice were associated with Nrf2-regulation of oxidative stress and GSH metabolism pathways (Thomas et al., 2013a), an observation also consistent with GSH loss during CP exposures.

Metabolite Model Parameterization: The rate equations for the three components of the expanded model, i.e., 1-CEO (2A), RP (2B) and GSH (2C) are in Figure 2. The parameters used in the CP metabolite submodel are shown in Table 1 (IVIVE scaling of *in vitro* derived rate constants was performed in the same manner as in the parent chemical model as discussed above). The fraction of total CP metabolism to 1-CEO in liver (ALPHAL) and lung (ALPHALU) was reported in Himmelstein et al. (2004) for female mouse and rat. The fraction of 1-CEO that is available for distribution, hydrolysis, or oxidative metabolism (BETA) was set equal to the ratio for epoxybutene (Campbell et al., 2015) where 67% of the amount of epoxybutene produced from the metabolism of butadiene was further metabolized due to co-localization of enzymes (i.e., CYP P450 and EH) in the endoplasmic reticulum. The *in vitro* derived parameters for the hydrolysis and oxidative (mouse only) metabolism of 1-CEO in liver and lung were reported in Himmelstein et al. (2004). For the oxidative pathway in mouse, only the male mouse liver incubations provided levels of metabolism that allowed estimation of the 1-CEO saturable metabolism parameters. The oxidative metabolism of 1-CEO in the lung of the mouse was not measurable. Since the incubations with mouse lung microsomes did not allow for saturable metabolism parameter estimation of 1-CEO (Clewell et al., 2019), the maximum rate (VMAX1O) for the mouse lung was based on the lung to liver metabolism ratio (LLOXACT) previously estimated for the mouse using *in vitro* metabolism data (Clewell et al., 2019). The chemical reaction rate constants for the RPs, including the second order reaction with GSH in liver (K2L) and lung (K2LU), and reaction rate with other cellular molecules (KFEE) were taken from Clewell et al. (2001). For KFEELU, a scaler (LLEEACT) was based on the liver to lung activity of glutathione *S*-transferase (Environ International, 2004 using activities reported by Andersen et al., 1987 and GSH parameters for liver and lung estimated from D’Souza et al., 1988). The partition coefficients for 1-CEO were calculated with the IndusChemFate model (ver. 2.0). Simulations with the CP model were carried out in R (ver. 4.0.3). The metabolite submodel code is included in the Supplementary Materials along with the physiological parameters (Supplementary Table S2) and partition coefficients (Supplementary Table S3) for the chloroprene model.

**TABLE 1:** Parameters for the CP metabolite model (physiological parameters and chloroprene partition coefficients are reported in supplemental materials).

Parameter	Description	Female mouse	Female rat
Chloroprene			
ALPHAL	Fraction of oxidative metabolism to 1-CEO in liver (remainder to 2-CEO)	0.02^a^	0.05^a^
ALPHALU	Fraction of oxidative metabolism to 1-CEO in lung (remainder to 2-CEO)	0.03^a^	0.15^a^
Fraction of total CP to 1-CEO privileged access			
BETA	Fraction of 1-CEO production available for hydrolysis/oxidative metabolism or release to blood	0.33^b^	0.33^b^
1-CEO			
Metabolism in Liver—Hydrolysis			
VMAXC1	Scaled VMax for Hydrolysis Pathway:Liver (mg/h/BW^0.75)	10.65^a^	62.1^a^
KM1	Km for Hydrolysis Pathway:Liver (mg/L)	1.9^a^	3.7^a^
Metabolism in Lung—Hydrolysis			
VMAXCLU1	Scaled VMax for Hydrolysis Pathway:Lung (mg/h/BW^0.75)	0.64^a^	0.85^a^
KMLU1	Km for Hydrolysis Pathway:Lung (mg/L)	4.6^a^	8.0^a^
Metabolism in Liver—Oxidative (Mouse pathway only)			
VMAXC1O	Scaled VMax for oxidative pathway in liver (mg/h/BW^0.75)	2.25^a^	NA
KM1O	Km for oxidative pathway in liver (mg/L)	1.5^a^	NA
LLOXACT	Lung to liver ratio for oxidative metabolism of 1-CEO (VMAXC10 scaled to lung)	0.42^c^	NA
Reactive Products			
KGSHLC	2nd order rate of RP reaction with GSH in liver (L/µmol/hr)	0.13^d^	0.13^d^
KGSHLUC	2nd order rate of RP reaction with GSH in lung (L/µmol/hr)	0.13^d^	0.13^d^
KFEEC	Conjugation rate with non-GSH (L/µmol/hr)	35^d^	35^d^
LLEEACT	Lung to liver ratio for reactive products reaction with other cellular molecules (KFEEC scaled to lung)	0.14^e^	0.06^e^
GSH Parameters from ECD model			
KPC	First-order rate constant for GSH production/loss (/hr*kg BW^-0.3)	0.06^f^	0.06^f^
GSO	Initial GSH concentration in liver (µM)	7,000^f^	5,500^f^
GSOLU	Initial GSH concentration in lung (µM)	1,500^f^	1,200^f^
1-CEO Partition Coefficients			
PB1	Blood:Air	5.74^g^	5.74^g^
PLU1	Lung:Blood	0.69^g^	0.69^g^
PL1	Liver:Blood	1.18^g^	1.18^g^
PF1	Fat:Blood	5.15^g^	5.15^g^
PS1	Slowly Perfused:Blood	0.69^g^	0.69^g^
PR1	Rapidly Perfused:Blood	1.18^g^	1.18^g^

^a^ Himmelstein et al., 2004.

^b^ Campbell et al., 2015.

^c^ LLOXACT, was set to ratio of lung to liver VMAXC, for mouse (Clewell et al., 2019).

^d^ Clewell et al., 2001.

^e^ LLEEACT, was set to ratio of lung to liver glutathione S-transferase for mouse and rat (Andersen et al., 1987).

^f^ D’Souza et al. (1988), Environ International (2004).

^g^ 1-CEO tissue:air and tissue:blood partitions were estimated using IndusChemFate (version 2.00, http://cefic-lri.org/toolbox/induschemfate/) and a logKow of 1.22 (KOWIN v.1.67 reported on Chemspider 2021. http://www.chemspider.com/Chemical-Structure.201536.html).

### 2.1 Sensitivity analysis

A one-at-a-time (OAT) forward-difference sensitivity analysis was conducted to determine which model parameters had the greatest influence on the response variable. The sensitivity of all model parameters (excluding BW, QCC, and QPC) was assessed for the three bioassay exposure levels (12.8, 32, and 80 ppm) for the dose metrics (amount CP metabolized/g lung/day, average concentration of RP in the lung and average concentration of 1-CEO). The sensitivity was assessed at the end of the second week of exposure (6 h/day for 5 days/week). Normalized sensitivity coefficients (fractional change in output divided by fractional change in input) were calculated. Normalization for the response variable and the parameter was included to allow a comparison across parameters and doses. The output was deemed sensitive to a parameter if the resulting coefficient was >0.1 in absolute value. Only parameters that were influential on at least one output metric are reported in supplemental (Supplementary Table S4 for female mouse and Supplementary Table S5 for female rat).

### 2.2 Uncertainty analysis

The uncertainty in predicted dose metrics (Tmet, Preact and 1-CEO) were assessed using Monte Carlo (MC) analysis where all model parameters were given defined distributions and sampled independently a large number of times to generate a stable distribution of the dose metrics. The distributions used for each parameter are given in Supplementary Table S6. The CV values chosen were previously reported by Clewell and Jarnot (1994) with coefficients of variation reflective of the relativity uncertainty in the model parameter. The simulation used for the dose metric comparisons reported above was retained (i.e., bioassay concentrations, 6 h/day, 5 days/week for 14 days) and 1,000 iterations of the model were captured for the dose metric summaries reported in Supplementary Table S7.

## 3 Results

**FIGURE 3:**
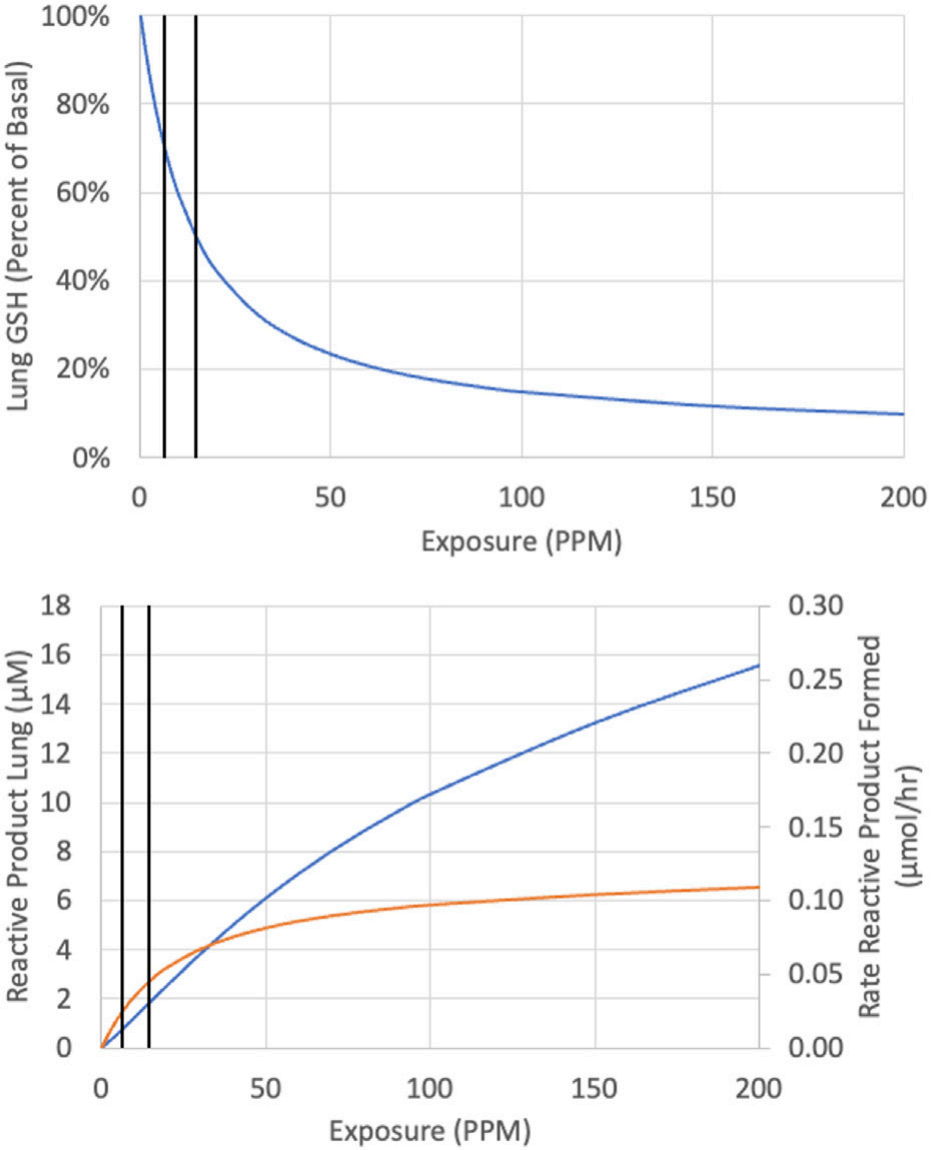
Predicted concentration of GSH (top panel) reactive product (bottom panel, blue line) and rate of reactive product formation (bottom panel, orange line) at the end of a single 6-h exposure to CP. Vertical black lines represent 6.3 and 14.6 ppm.

**FIGURE 4:**
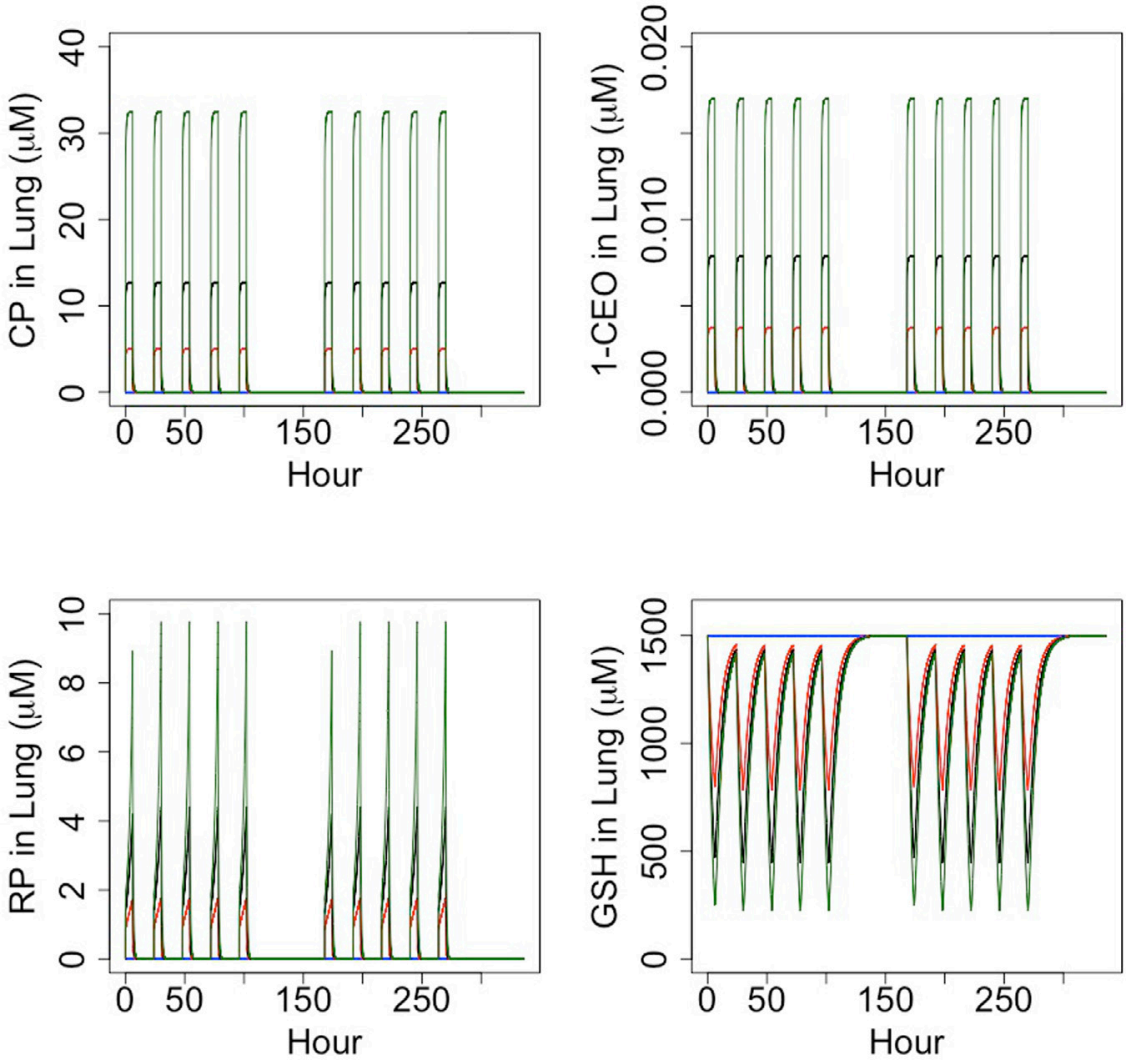
Timecourse simulation (6 h/day, 5 days/week) of the concentration of CP, 1-CEO, RP and GSH in female mouse lung for the CP bioassay exposure concentrations: control (blue), 12.8 ppm (red), 32 ppm (black) and 80 ppm (green).

Curves were first generated using the reactive-metabolite model to show the relationships between inhaled CP and expected GSH at the end of 6 h exposures and between inhaled CP and concentrations of reactive RPs and GSH at the end of 6 h (Figure 3). The rate of metabolism versus inhaled CP follows a Michaelis-Menten form, quickly approaching a maximum rate at several 100 ppm. The RPs formed by oxidation would deplete GSH, with depletion to about 50% of the initial value at 15.3 ppm. As GSH becomes depleted, RPs cannot be cleared as efficiently, and the RP concentration rises in a non-linear fashion with increasing exposure concentrations of CP. The relationship between CP, 1-CEO, RP and GSH in the lung is further illustrated in the 14 days simulation of the bioassay concentrations (Figure 4). Where the turnover of CP, 1-CEO and RP is rapid after the onset and end of exposure.

The extended model of CP metabolism described above was exercised to evaluate three potential dose metrics for the lung toxicity and carcinogenicity of CP: 1) total lung metabolism per gram lung per day (Tmet), the dose metric used in Clewell et al. (2019), 2) average concentration of RPs of metabolism in the lung (Preact), and 3) average concentration of 1-CEO in the lung (1-CEO).

**FIGURE 5:**
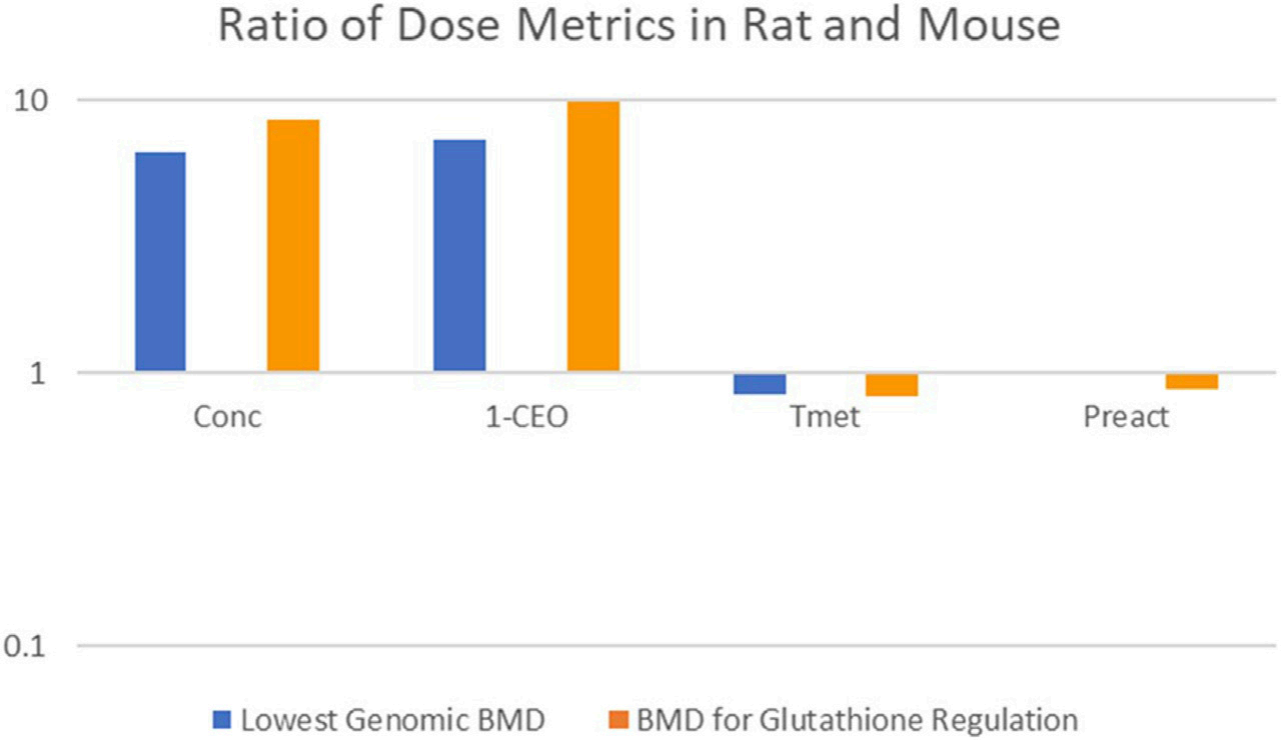
Cross-Species Consistency of CP Dose Metrics Based on Equivalence of Dose-Response for Gene Expression Changes (Thomas et al., 2013a). Bars represent the ratio (rat value divided by mouse value) of the lung dose metric values associated with each of the BMDs in the two species (Conc: inhaled concentration; 1-CEO: 1-chloroethenyl oxirane; Tmet: total metabolism of chloroprene per gram tissue; Preact: concentration of reactive products). No blue bar is visible for the Preact dose metric because the rat and mouse dose metrics were nearly identical.

The first comparison performed was an evaluation of the consistency of the alternative dose metrics with the gene expression dose-response data reported in Thomas et al. (2013a). In this study, female mice and rats were exposed to CP by inhalation 6 h per day, for 5 or 15 days. Mice were exposed to a range of concentrations (0.3, 12.8, 32, or 80 ppm) that were similar to those in the NTP (1998) bioassay, but a higher concentration range was used in the rat (5, 30, 90, or 200 ppm) to provide similar tissue doses based on predicted total amount of CP metabolized per gram of lung tissue per day from a preliminary version of the PBPK model of Yang et al. (2012). For this comparison, two genomic responses were used: the lowest Benchmark Dose (BMD) for any gene expression change and the lowest BMD for any gene expression change related to regulation of GSH homeostasis. It has previously been demonstrated that the transcriptional BMD values based on the most sensitive pathway are highly correlated with BMD values based on traditional apical responses for both noncancer (toxicity) and cancer-related (tumor) endpoints (Thomas et al., 2013b). Results of the comparison are shown in Figure 5, which displays the ratio of the lung dose metric values associated with each of the BMDs in the two species (rat value divided by mouse value). A successful dose-metric for cross-species extrapolation should predict that cellular responses in the lung begin to occur at similar values of the dose metric in both rats and mice, resulting in a ratio close to unity. However, the lowest BMD and the lowest BMD for glutathione regulation in the rat occur at nearly 10-fold higher inhaled concentrations (Conc) and 1-CEO concentrations (1-CEO) compared to the mouse. In contrast, they occur at similar values of total metabolism (Tmet) and reactive product concentration (Preact) in the two species, supporting the appropriateness of these dose metrics for cross-species extrapolation.

Consistent with the expectations that drove the experimental design in Thomas et al. (2013a), the inhaled concentrations of CP at which there was genomic evidence of cellular stress in the lungs of the rat were much higher than in the mouse. The predicted dose metric values for 1-CEO concentration associated with similar genomic biomarkers of cellular effects are also nearly an order of magnitude higher in the rat than in the mouse. In contrast, the model predicts similar dose metric values for both Tmet and Preact in the rat and mouse, consistent with the expectation that cellular responses to CP in the lung would begin to occur at similar levels of cellular stress, i.e., similar levels of GSH depletion. The consistency of these two-dose metrics with the observed genomic dose-response in the female mouse and female rat, and the inconsistency of the 1-CEO or inhaled CP dose metrics, support the importance of RP formation and GSH depletion in the mode of action for CP.

**FIGURE 6:**
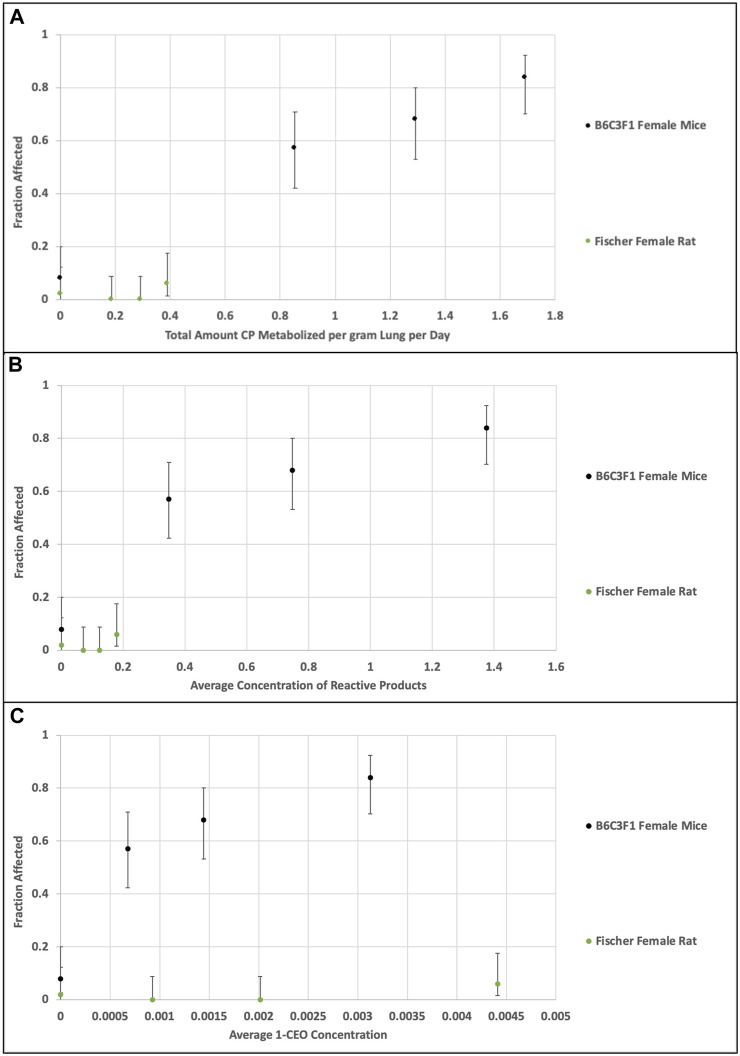
Comparison of dose metrics in rats and mice **(A)**—total amount CP metabolized per gram lung per day; **(B)** average concentration of RPs in lung; **(C)** average 1-CEO concentration in lung) predicted with the CP model for the NTP (1998) bioassay inhaled CP concentrations (0, 12.8, 32, or 80 ppm, 14 days, 6 h/day, 5 days/week). Whereas the relationship of the dose metrics for total metabolism and reactive product formation in rat and mouse are consistent with the observed tumor incidence, the dose metrics for 1-CEO in the nonresponsive rat are actually greater than those associated with high tumor incidence in the mouse.

The second comparison performed was an evaluation of the consistency of the alternative dose metrics, total CP metabolism per gram liver per day, average RP concentration and average 1-CEO concentration, with the tumor incidence in the bioassays for the female mouse and rat. Figures 6A–C plots the predicted dose metrics (Tmet, Preact and 1-CEO) against the tumor incidence in the female mouse and female rat in the NTP (1998) bioassays (0, 12.8, 32, or 80 ppm). As in the previous comparison of genomic responses after short-term exposures, the Tmet and Preact metrics provide a reasonable dose-response relationship with tumor incidence, whereas the 1-CEO metric does not. In fact, as shown in Figures 5, 6C, using the 1-CEO concentration as the dose metric would predict that the female rat should have had a higher tumor incidence than the female mouse, and this was not observed in the NTP (1998) bioassay.

The inconsistency of the 1-CEO dose metric with the relationships for both toxicity and carcinogenicity between the female mouse and female rat is likely due to the small proportion of total CP metabolism that it represents. At the bioassay concentrations, the predicted concentrations of 1-CEO are less than 0.4% of the concentrations of RPs in the female mouse and less than 5% in the rat.

The normalized sensitivity coefficients for the dose metrics (Tmet: total CP metabolized per gram lung per day, Preact: average concentration reactive product and 1-CEO: average concentration of 1-CEO) and the average concentration of lung GSH (CGSH) for the three bioassay concentrations are shown in Supplementary Table S4, S5 (female mouse) and (female rat). The results are as expected where parameters directly related to the production or loss as well as physiological constants related to uptake and distribution were sensitive to the respective dose metric.

Uncertainty in the dose metrics (Tmet, Preact and 1-CEO) evaluated from the MC simulation is shown in Supplementary Table S7. For Tmet, the dose metric proposed by Clewell et al. (2019), and 1-CEO (average concentration of 1-CEO), the uncertainty is relatively stable across the three exposure levels with CVs of ∼40–46%. For the average concentration of reactive products (Preact), the uncertainty is approximately four-fold higher (CV: 230% to 160%).

## 4 Discussion

The toxicology and metabolism of both vinyl chloride (VC) and vinylidene chloride (VDC; 1,1-dichloroethylene) have been extremely well-characterized due to their uses as precursors for a variety of polymeric products. The research on toxicity of these compounds, which dates to the early 1970s, initially focused more on effects in the liver rather than effects on the lung, largely due to the association of VC with increased liver hemangiosarcoma in workers exposed to high concentrations of VC (Makk et al., 1974). As with CP, the pathways of metabolism involve 1) CYP P450 oxidation, 2) production of reactive intermediates, 3) reaction of these reactive metabolites with GSH and, 4), after sufficient GSH depletion, reaction with other cellular constituents. In the case of VC, there is also evidence of a genotoxic component. VC exposure results in liver angiosarcoma in mouse, rat, hamster and human at inhaled concentrations well below those associated with toxicity. The mode of action for liver angiosarcoma from VC has been suggested to result from the metabolic production in hepatocytes of an epoxide (chloroethylene epoxide) that is sufficiently stable to diffuse into adjacent sinusoidal cells (Laib and Bolt, 1980). Significantly, whereas the epoxide of VC has a half-life of 1.6 min (Plugge and Safe, 1977), CP and l,l-DCE are expected to have short-lived epoxides (Plugge and Jaeger, 1979), consistent with the results of the PBPK modelling reported here (Figure 4). From an analytical perspective, these transient metabolites react so quickly that their concentrations during *in vivo* exposures cannot be directly determined.

Liver GSH levels are lower in fasted rats than in fed rats (Jaeger et al., 1974). With this reduction in GSH, compounds that deplete liver GSH are more toxic to fasted than to fed rats. The LC50 of 1,1-DCE in fed rats was 15,000 ppm, but in fasted rats it was only 150 ppm. Serum enzymes increased abruptly at 100 ppm and were maximum at several hundred ppm. These responses are due to production of reactive metabolites that are cleared by GSH until the GSH becomes depleted. While the initial oxidation of 1,1-DCE produces an epoxide, this metabolite is unstable and undergoes spontaneous rearrangement most likely producing chloroacetyl chloride, a highly reactive acid halide. These metabolites react with and deplete GSH levels. With severe GSH depletion, these metabolites react with tissue constituents leading to macromolecular binding and tissue toxicity (McKenna et al., 1977). Unlike VC, 1,1-DCE does not cause significant increases in hemangiosarcoma or any other liver tumors. However, with both VC and VDC, all metabolism goes through a single epoxide.

In early work examining the hepatic toxicity of CP in rats including the effects of fasting to restrict GSH resynthesis, Plugge and Jaeger (1979) noted that the pattern of toxicity was comparable to VDC although higher exposures of CP were required to produce equivalent increases in serum ALT (Jaeger et al., 1974; Plugge and Jaeger, 1979). As noted earlier, metabolism of CP mainly produces a combination of reactive aldehydes and ketones derived from 2-(chloroethenyl)-oxirane with only 3% of total metabolism in female mouse lung producing 1-CEO (α*β, Figure 1A) which is further oxidized to reactive products by a second oxidation in the mouse. Using kinetic constants determined for GSH synthesis and consumption from studies with styrene and styrene oxide, the model demonstrates that the metabolism of CP in the lungs is expected to cause depletion of GSH (Figure 3), consistent with the observation that lung transcriptomic responses indicative of changes in GSH metabolism are the most sensitive ontology pathway (Thomas et al., 2013a). Only with sufficient GSH depletion will the reactivity with tissue components lead to extensive macromolecular binding and overt toxicity and increased tumor incidence. With VC, it was estimated that there was relatively little macromolecular binding if depletion was less than 30%. Here, our analysis for CP showed that tumor incidence tracks with total metabolized or expected concentration of RPs rather than inhaled CP or 1-CEO concentrations. All the bioassay concentrations in the mouse or rat (12.8, 30 and 80 ppm) are expected to cause much more than 30% depletion of GSH from basal levels (Figures 3, 4). Depletion of GSH 30% below basal levels is predicted to occur following inhalation of 6.3 ppm and a 50% reduction in GSH following inhalation of 14.6 ppm in female mice. Our modeling results capture the non-linear relationship between RPs and total rate of metabolism (Figure 3). These results demonstrate the continued increase in RPs as the exposure increases, despite the prediction that RP production has been saturated, a prediction that is consistent with the body of work on the toxicology of these chlorinated compounds as well as with the dose response for tumors observed following inhalation exposure of mice or rats to CP.

Both 1-CEO and, (Z)-2-chlorobut-2-en-1-al, a reactive aldehyde derived from 2-CEO, formed adducts when incubated with specific nucleotides (Munter et al., 2007). While the studies are not necessarily representative of reaction conditions with native DNA *in vivo*, they show the ability of 1-CEO and at least one of the reactive CP metabolites to react with bases in DNA and form adducts. Our MOA with CP does not dismiss formation of these adducts but instead highlights that there is a threshold below which macromolecular binding is small and the cancer dose response is driven by production of reactive metabolites together with increasing levels of GSH depletion. Small changes in the numbers of adducts are not expected to define the shape of the dose response curve at low doses. In fact, there is always a substantial background of various adducts with more than 40,000 altered bases per cell (Nakamura et al., 2014). At low levels of exposure, with increases in only a small number of adducts, DNA damage response networks would still be capable of effectively maintaining the integrity of the DNA prior to cell division through non-linear feedback processes (Zhang et al., 2014; Clewell and Andersen, 2016).

Overall, the dose response for lung tumors from CP is consistent with a non-linear cytotoxic MOA, with macromolecular binding to protein, lipid and nucleic acid bases increasing non-linearly at higher exposures that are also expected to deplete lung GSH.

## Figures and Tables

**Table:** 

**1-CEO**	(1-chloroethenyl)oxirane (1-CEO)
**1,1-DCE**	1,1-Dichloroethylene
**2-CEO**	(2-chloroethenyl)oxirane (2-CEO)
**ALPHAL**	Fraction of oxidative metabolism to 1-CEO in liver (remainder to 2-CEO)
**ALPHALU**	Fraction of oxidative metabolism to 1-CEO in lung (remainder to 2-CEO)
**BETA**	Fraction of 1-CEO production available for hydrolysis/oxidative metabolism or release to blood
**BMD**	Benchmark dose
**CP**	2-chloro-1,3-butadiene
**CYP**	cytochrome
**DNA**	Deoxy ribonucleic acid
**EDC**	1,2-Dichloroethane
**EH**	epoxide hydrolase (EH)
**GSO**	Initial GSH concentration in liver
**GSOLU**	Initial GSH concentration in lung
**GST**	glutathione transferase (GST)
**KFEEC**	Conjugation rate with non-GSH
**KGSHLC**	2nd order rate of RP reaction with GSH in liver
**KGSHLUC**	2nd order rate of RP reaction with GSH in lung
**KM1**	Km for Hydrolysis Pathway: Liver
**KM1O**	Km for oxidative pathway in liver
**KMLU1**	Km for Hydrolysis Pathway: Lung
**KPC**	First-order rate constant for GSH loss
**LLEEACT**	Lung to liver ratio for reactive products reaction with other cellular molecules (KFEEC scaled to lung)
**LLOXACT**	Lung to liver ratio for oxidative metabolism of 1-CEO (VMAXC10 scaled to lung)
**MOA**	Mode of Action
**MPPGL**	Microsomal protein per gram liver or lung
**NADP+/NADPH**	Nicotinamide adenine dinucleotide phosphate
**PBPK**	Physiologically Based Pharmacokinetic Model
**Preact**	Average concentration reactive product in lung
**RP**	Reactive product
**Tmet**	Total amount CP metabolized per gram lung per day
**VC**	Vinyl chloride
**VDC**	Vinylidene chloride
**VMAXC1**	Scaled VMax for Hydrolysis Pathway: Liver
**VMAXC1O**	Scaled VMax for oxidative pathway in liver
**VMAXCLU1**	Scaled VMax for Hydrolysis Pathway: Lung

## Data Availability

The original contributions presented in the study are included in the article/Supplementary Material, further inquiries can be directed to the corresponding author.
