# Supplementary material for: Using available in vitro metabolite identification and time course kinetics for β-chloroprene and its metabolite, (1-chloroethenyl) oxirane, to include reactive oxidative metabolites and glutathione depletion in a PBPK model for β-chloroprene
Source: Front Pharmacol. 2023 Aug 17;14:1223808. doi: 10.3389/fphar.2023.1223808 (PMC10472072; doi:10.3389/fphar.2023.1223808)
Supplement: Supplementary file 1 [file DataSheet1.DOCX]

Supplementary Material

Using available *in vitro* metabolite identification and time course kinetics for β-chloroprene and its metabolite, (1-chloroethenyl)oxirane, to include reactive oxidative metabolites and glutathione depletion in a PBPK model for β-chloroprene

Campbell JL*, Clewell HJ III, Van Landingham C, Gentry PR, Andersen ME

*** Correspondence:** Corresponding Author: [jcampbell@ramboll.com](mailto:jcampbell@ramboll.com)

| **Table S1: Average adult parameters recommended for in vitro to in vivo extrapolation** | | | |  |
| --- | --- | --- | --- | --- |
| **Parameter** | **Female Mouse** | **Female Rat** | **Reference** |  |
| **BW (kg)** | 0.035 | 0.33 | Brown et al, 1997 (page 415 in text) |  |
|  |  |  |  |  |
| **Liver fractional weight (VLC)** | 0.0549 | 0.0366 | Brown et al, 1997 (Tables 4, 5) |  |
|  |  |  |  |  |
| **Lung fractional weight (VLUC)** | 0.0073 | 0.005 | Brown et al, 1997 (Tables 4, 5) |  |
|  |  |  |  |  |
| **Kidney fractional weight (VKC)** | 0.0167 | 0.0073 | Brown et al, 1997 (Tables 4, 5) |  |
|  |  |  |  |  |
| **Liver mg microsomal protein per g liver (MPPGL)** | 35 | 40 | Medinsky et al., 1994 for mouse; Medinsky et al., 1994 and Houston and Galetin, 2008 for rat |  |
|  |  |  |  |  |
| **Lung mg microsomal protein per g lung (MPPGLU)** | 20 | 20 | Medinsky et al., 1994 and Boogard et al., 2000 for all species |  |
|  |  |  |  |  |
| **Kidney mg microsomal protein per g kidney (MPPGK)b** | 18 | 18 | Yoon et al., 2007 for mouse and rat |  |
|  |  |  |  |  |

| **Table S2: Physiological Parameters for CP PBPK Model** | | | | | | |  |
| --- | --- | --- | --- | --- | --- | --- | --- |
| **Parameter** | **Abbreviation** | **Units** | **Female Mouse** | **Source** | **Female Rat** | **Source** |  |
| Body Weight | BW | kg | 0.04 | NTP (1998) time and survival weighted average BW control animals | 0.256 | NTP (1998) time and survival weighted average BW control animals |  |
| Alveolar Ventilation | QPC | L/h/kg^0.75^ | 29.1 | Brown et al. 1997 (Table 31) | 22.4 | Brown et al. 1997 (Table 31) |  |
| Cardiac Output | QCC | L/h/kg^0.75^ | 20.1 | Marino et al. 2006  (QPC/QCC = 1.45) | 18.7 | Brown et al. 1997 (Table 22) |  |
| **FRACTIONAL BLOOD FLOWS TO TISSUES** | | | | | | |  |
| Flow to Liver as fraction Cardiac Output | QLC | unitless | 0.161 | Brown et al. 1997 (Table 23) | 0.183 | Brown et al. 1997 (Table 23) |  |
| Flow to Fat as fraction Cardiac Output | QFC | unitless | 0.07 | Brown et al. 1997 (Table 23; Same as rat value) | 0.07 | Brown et al. 1997 (Table 23) |  |
| Flow to Slow as fraction Cardiac Output | QSC | unitless | 0.159 | Brown et al. 1997 (Table 23); Same as that reported for muscle | 0.278 | Brown et al. 1997 (Table 23); Same as that reported for muscle |  |
| Flow to Kidney as fraction Cardiac Output | QKC | unitless | 0.09 | Brown et al. 1997 (Table 23) | 0.14 | Brown et al. 1997 (Table 23) |  |
| **FRACTIONAL VOLUMES OF TISSUES** | | | | | | |  |
| Volume Liver as fraction Body Weight | VLC | unitless | 0.0549 | Brown et al. 1997 (Table 4) | 0.0366 | Brown et al. 1997 (Table 5) |  |
| Volume Lung as fraction Body Weight | VLUC | unitless | 0.0073 | Brown et al. 1997 (Table 4) | 0.005 | Brown et al. 1997 (Table 5) |  |
| Volume Fat as fraction Body Weight | VFC | unitless | 0.1 | Brown et al. 1997 (Table 10) | 0.1 | Brown et al. 1997 (Table 13) |  |
| Volume Rapid Perfused as fraction Body Weight | VRC | unitless | 0.08098 | Brown et al. 1997 (Table 4); Sum of adrenals, brain, stomach, small intestine, large intestine, heart, lungs, pancreas, spleen and thyroid | 0.04644 | Brown et al. 1997 (Table 5); Sum of adrenals, brain, stomach, small intestine, large intestine, heart, lungs, pancreas, spleen and thyroid |  |
| Volume Slow Perfused as fraction Body Weight | VSC | unitless | 0.384 | Brown et al. 1997 (Table 4); Same as that reported for muscle | 0.4 | Brown et al. 1997 (Table 5); Same as that reported for muscle |  |
|  |  |  |  |  |  |  |  |
| Volume Kidney as fraction Body Weight | VKC | unitless | 0.0167 | Brown et al. 1997 (Table 4) | 0.0073 | Brown et al. 1997 (Table 5) |  |

| **Table S3: Partition Coefficients for CP (Himmelstein et al. 2004)** | | |
| --- | --- | --- |
|  | **Mice** | **Rats** |
| Blood:Air | 7.83 | 7.35 |
| Lung:Blood | 2.38 | 1.85 |
| Liver:Blood | 1.26 | 1.58 |
| Fat:Blood | 17.35 | 16.99 |
| Muscle:Blood ^a^ | 0.59 | 0.60 |
| Kidney:Blood ^b^ | 1.76 | 2.29 |
| ^a^ used for slowly perfused tissues  ^b^ used for rapidly perfused tissues | | |

| **Table S4: Normalized sensitivity coefficients for female mouse assessed at the end of 14 days of simulation (6 hours/day, 5 days/week)** | | | | | | | | | | | | | | | |
| --- | --- | --- | --- | --- | --- | --- | --- | --- | --- | --- | --- | --- | --- | --- | --- |
|  |  | **12.8 ppm** | |  |  |  | **32 ppm** |  |  |  |  | **80 ppm** |  |  |  |
| **Parameter** | **Tmet** | **Preact** | **1-CEO** | **CGSH** |  | **Tmet** | **Preact** | **1-CEO** | **CGSH** |  | **Tmet** | **Preact** | **1-CEO** | **CGSH** |  |
| **BW** | -0.2 | -0.2 |  |  |  | -0.2 | -0.3 |  |  |  | -0.2 | -0.3 |  |  |  |
| **QPC** | 0.3 | 0.4 |  |  |  |  | 0.3 |  |  |  |  | 0.2 |  |  |  |
| **QCC** | -0.2 | -0.4 | 0.4 |  |  |  | -0.3 | 0.6 |  |  |  | -0.3 | 0.8 |  |  |
| **QLC** | -0.2 | -0.3 | 0.4 |  |  |  | -0.3 | 0.5 |  |  |  | -0.2 | 0.7 |  |  |
| **QFC** |  |  |  |  |  |  |  |  |  |  |  |  |  |  |  |
| **QRC** |  |  | -0.2 |  |  |  |  | -0.3 |  |  |  |  | -0.4 |  |  |
| **QSC** |  |  |  |  |  |  |  |  |  |  |  |  |  |  |  |
| **QKC** |  |  |  |  |  |  |  |  |  |  |  |  |  |  |  |
| **VLC** |  |  |  |  |  |  |  |  |  |  |  | 0.2 |  |  |  |
| **VLUC** | -1.0 | -1.4 |  |  |  | -1.0 | -1.9 |  | 0.2 |  | -1.0 | -2.9 |  | 0.3 |  |
| **VFC** |  | 0.2 |  |  |  |  | 0.4 |  |  |  | 0.2 | 0.6 |  |  |  |
| **VRC** |  |  |  |  |  |  | 0.3 |  |  |  |  | 0.4 |  |  |  |
| **VSC** | 0.6 | 0.8 |  |  |  | 0.6 | 1.2 |  |  |  | 0.6 | 1.8 |  |  |  |
| **VKC** |  |  |  |  |  |  |  |  |  |  |  |  |  |  |  |
| **PL** |  |  |  |  |  |  |  |  |  |  |  |  |  |  |  |
| **PLU** |  |  |  |  |  |  |  |  |  |  |  |  |  |  |  |
| **PF** |  |  |  |  |  |  |  |  |  |  |  |  |  |  |  |
| **PS** |  |  |  |  |  |  |  |  |  |  |  |  |  |  |  |
| **PR** |  |  |  |  |  |  |  |  |  |  |  |  |  |  |  |
| **PB** | 0.3 | 0.4 | 0.4 |  |  | 0.2 | 0.4 | 0.4 |  |  |  | 0.4 | 0.5 |  |  |
| **PK** |  |  |  |  |  |  |  |  |  |  |  |  |  |  |  |
| **PL1** |  |  |  |  |  |  |  |  |  |  |  |  |  |  |  |
| **PLU1** |  |  | 1.0 |  |  |  |  | 1.0 |  |  |  |  | 1.0 |  |  |
| **PF1** |  |  |  |  |  |  |  |  |  |  |  |  |  |  |  |
| **PS1** |  |  |  |  |  |  |  |  |  |  |  |  |  |  |  |
| **PR1** |  |  |  |  |  |  |  |  |  |  |  |  |  |  |  |
| **PB1** |  |  | 0.5 |  |  |  |  | 0.6 |  |  |  |  | 0.6 |  |  |
| **ALPHAL** |  |  | 0.6 |  |  |  |  | 0.7 |  |  |  |  | 0.8 |  |  |
| **ALPHALU** |  |  | 0.4 |  |  |  |  | 0.3 |  |  |  |  |  |  |  |
| **BETA** |  |  | 1.0 |  |  |  |  | 1.0 |  |  |  |  | 1.0 |  |  |
| **VMAXC** |  |  |  |  |  |  |  |  |  |  |  |  |  |  |  |
| **KM** |  |  |  |  |  |  |  |  |  |  |  |  |  |  |  |
| **VMAXCLU** | 1.0 | 1.4 | 0.4 |  |  | 1.0 | 2.1 | 0.3 | -0.2 |  | 1.0 | 3.2 |  | -0.3 |  |
| **KMLU** | -0.5 | -0.8 | -0.2 |  |  | -0.3 | -0.7 |  |  |  |  | -0.6 |  |  |  |
| **VMAXC1** |  |  | -0.5 |  |  |  |  | -0.6 |  |  |  |  | -0.6 |  |  |
| **KM1** |  |  | 0.4 |  |  |  |  | 0.4 |  |  |  |  | 0.5 |  |  |
| **VMAXCLU1** |  |  |  |  |  |  |  |  |  |  |  |  |  |  |  |
| **KMLU1** |  |  |  |  |  |  |  |  |  |  |  |  |  |  |  |
| **VMAXC1O** |  |  | -0.2 |  |  |  |  | -0.2 |  |  |  |  | -0.2 |  |  |
| **KM1O** |  |  | 0.2 |  |  |  |  | 0.2 |  |  |  |  | 0.2 |  |  |
| **KGSHLC** |  |  |  |  |  |  |  |  |  |  |  |  |  |  |  |
| **KGSHLUC** |  | -0.9 |  |  |  |  | -0.9 |  |  |  |  | -0.8 |  |  |  |
| **LLOXACT** |  |  |  |  |  |  |  |  |  |  |  |  |  |  |  |
| **LLEEACT** |  |  |  |  |  |  |  |  |  |  |  | -0.2 |  |  |  |
| **KFEEC** |  |  |  |  |  |  |  |  |  |  |  | -0.2 |  |  |  |
| **KPC** |  | -0.2 |  |  |  |  | -0.5 |  | 0.3 |  |  | -1.0 |  | 0.3 |  |
| **GSO** |  |  |  |  |  |  |  |  |  |  |  |  |  |  |  |
| **GSOLU** |  | -1.3 |  | 1.1 |  |  | -1.9 |  | 1.2 |  |  | -2.7 |  | 1.3 |  |

| **Table S5: Normalized sensitivity coefficients for female rat assessed at the end of 14 days of simulation (6 hours/day, 5 days/week)** | | | | | | | | | | | | | | |
| --- | --- | --- | --- | --- | --- | --- | --- | --- | --- | --- | --- | --- | --- | --- |
|  |  | **12.8 ppm** |  |  |  |  | **32 ppm** |  |  |  |  | **80 ppm** |  |  |
| **Parameter** | **Tmet** | **Preact** | **1-CEO** | **CGSH** |  | **Tmet** | **Preact** | **1-CEO** | **CGSH** |  | **Tmet** | **Preact** | **1-CEO** | **CGSH** |
| **BW** | -0.2 |  |  |  |  | -0.2 |  |  |  |  | -0.22 |  |  |  |
| **QPC** | 0.3 | 0.3 |  |  |  |  | 0.2 |  |  |  |  |  |  |  |
| **QCC** | -0.3 | -0.3 | 0.3 |  |  | -0.2 | -0.3 | 0.5 |  |  |  | -0.22 | 0.67 |  |
| **QLC** | -0.3 | -0.3 | 0.3 |  |  |  | -0.2 | 0.5 |  |  |  |  | 0.58 |  |
| **QFC** |  |  |  |  |  |  |  |  |  |  |  |  |  |  |
| **QRC** |  |  |  |  |  |  |  |  |  |  |  |  | -0.23 |  |
| **QSC** |  |  |  |  |  |  |  |  |  |  |  |  |  |  |
| **QKC** |  |  |  |  |  |  |  |  |  |  |  |  |  |  |
| **VLC** |  |  |  |  |  |  |  |  |  |  |  |  |  |  |
| **VLUC** | -1.0 | -1.0 |  |  |  | -1.0 | -1.1 |  |  |  | -0.98 | -1.14 |  |  |
| **VFC** |  |  |  |  |  | 0.2 | 0.2 |  |  |  | 0.26 | 0.30 |  |  |
| **VRC** |  |  |  |  |  |  |  |  |  |  |  |  |  |  |
| **VSC** | 0.7 | 0.7 |  |  |  | 0.6 | 0.7 |  |  |  | 0.60 | 0.70 |  |  |
| **VKC** |  |  |  |  |  |  |  |  |  |  |  |  |  |  |
| **PL** |  |  |  |  |  |  |  |  |  |  |  |  |  |  |
| **PLU** |  |  |  |  |  |  |  |  |  |  |  |  |  |  |
| **PF** |  |  |  |  |  |  |  |  |  |  |  |  |  |  |
| **PS** |  |  |  |  |  |  |  |  |  |  |  |  |  |  |
| **PR** |  |  |  |  |  |  |  |  |  |  |  |  |  |  |
| **PB** | 0.3 | 0.3 | 0.4 |  |  | 0.2 | 0.3 | 0.4 |  |  |  | 0.21 | 0.43 |  |
| **PK** |  |  |  |  |  |  |  |  |  |  |  |  |  |  |
| **PL1** |  |  |  |  |  |  |  |  |  |  |  |  |  |  |
| **PLU1** |  |  | 1.0 |  |  |  |  | 1.0 |  |  |  |  | 1.00 |  |
| **PF1** |  |  |  |  |  |  |  |  |  |  |  |  |  |  |
| **PS1** |  |  |  |  |  |  |  |  |  |  |  |  |  |  |
| **PR1** |  |  |  |  |  |  |  |  |  |  |  |  |  |  |
| **PB1** |  |  | 0.5 |  |  |  |  | 0.5 |  |  |  |  | 0.53 |  |
| **ALPHAL** |  |  | 0.6 |  |  |  |  | 0.7 |  |  |  |  | 0.84 |  |
| **ALPHALU** |  |  | 0.4 |  |  |  | -0.2 | 0.3 |  |  |  | -0.22 |  |  |
| **BETA** |  |  | 1.0 |  |  |  |  | 1.0 |  |  |  |  | 1.00 |  |
| **VMAXC** |  |  |  |  |  |  |  |  |  |  |  |  |  |  |
| **KM** |  |  |  |  |  |  |  |  |  |  |  |  |  |  |
| **VMAXCLU** | 1.0 | 1.1 | 0.3 |  |  | 1.0 | 1.2 | 0.2 |  |  | 1.00 | 1.25 |  |  |
| **KMLU** | -0.6 | -0.6 | -0.2 |  |  | -0.4 | -0.5 |  |  |  | -0.24 | -0.30 |  |  |
| **VMAXC1** |  |  | -0.6 |  |  |  |  | -0.7 |  |  |  |  | -0.74 |  |
| **KM1** |  |  | 0.6 |  |  |  |  | 0.7 |  |  |  |  | 0.77 |  |
| **VMAXCLU1** |  |  |  |  |  |  |  |  |  |  |  |  |  |  |
| **KMLU1** |  |  |  |  |  |  |  |  |  |  |  |  |  |  |
| **KGSHLC** |  |  |  |  |  |  |  |  |  |  |  |  |  |  |
| **KGSHLUC** |  | -1.0 |  |  |  |  | -1.0 |  |  |  |  | -0.97 |  |  |
| **LLOXACT** |  |  |  |  |  |  |  |  |  |  |  |  |  |  |
| **LLEEACT** |  |  |  |  |  |  |  |  |  |  |  |  |  |  |
| **KFEEC** |  |  |  |  |  |  |  |  |  |  |  |  |  |  |
| **KPC** |  |  |  |  |  |  |  |  |  |  |  |  |  |  |
| **GSO** |  |  |  |  |  |  |  |  |  |  |  |  |  |  |
| **GSOLU** |  | -1.0 |  | 1.0 |  |  | -1.1 |  | 1.1 |  |  | -1.13 |  | 1.10 |

| **Table S6: Female mouse parameter distributions for uncertainty analysis** | | | | | | |
| --- | --- | --- | --- | --- | --- | --- |
| **Parameter** | **Value** | **CV** | **STD** | **Lower** | **Upper** | **Distribution** |
| **BW** | 4.00E-02 | 0.11 | 4.40E-03 | 3.12E-02 | 4.88E-02 | Normal |
| **QPC** | 2.91E+01 | 0.56 | 1.63E+01 | 4.66E+00 | 5.35E+01 | Normal |
| **QCC** | 2.01E+01 | 0.083 | 1.67E+00 | 1.68E+01 | 2.34E+01 | Normal |
| **QLC** | 1.61E-01 | 0.3 | 4.83E-02 | 6.44E-02 | 2.58E-01 | Normal |
| **QFC** | 7.00E-02 | 0.6 | 4.20E-02 | 2.80E-03 | 1.37E-01 | Normal |
| **QSC** | 1.59E-01 | 0.4 | 6.36E-02 | 3.18E-02 | 2.86E-01 | Normal |
| **QKC** | 9.00E-02 | 0.3 | 2.70E-02 | 3.60E-02 | 1.44E-01 | Normal |
| **QRC** | 5.20E-01 | 0.2 | 1.04E-01 | 3.12E-01 | 7.28E-01 | Normal |
| **VLC** | 5.50E-02 | 0.06 | 3.30E-03 | 4.84E-02 | 6.16E-02 | Normal |
| **VLUC** | 7.30E-03 | 0.3 | 2.19E-03 | 2.92E-03 | 1.17E-02 | Normal |
| **VFC** | 1.00E-01 | 0.3 | 3.00E-02 | 4.00E-02 | 1.60E-01 | Normal |
| **VRC** | 8.10E-02 | 0.3 | 2.43E-02 | 3.24E-02 | 1.30E-01 | Normal |
| **VSC** | 3.84E-01 | 0.3 | 1.15E-01 | 1.54E-01 | 6.14E-01 | Normal |
| **VKC** | 1.67E-02 | 0.3 | 5.01E-03 | 6.68E-03 | 2.67E-02 | Normal |
| **PL** | 1.26E+00 | 0.2 | 2.52E-01 | 8.31E-01 | 1.84E+00 | Log-Normal |
| **PLU** | 2.38E+00 | 0.2 | 4.76E-01 | 1.57E+00 | 3.47E+00 | Log-Normal |
| **PF** | 1.74E+01 | 0.3 | 5.21E+00 | 9.24E+00 | 2.99E+01 | Log-Normal |
| **PS** | 5.90E-01 | 0.2 | 1.18E-01 | 3.89E-01 | 8.60E-01 | Log-Normal |
| **PR** | 1.76E+00 | 0.2 | 3.52E-01 | 1.16E+00 | 2.56E+00 | Log-Normal |
| **PB** | 7.80E+00 | 0.2 | 1.56E+00 | 5.15E+00 | 1.14E+01 | Log-Normal |
| **PK** | 1.76E+00 | 0.2 | 3.52E-01 | 1.16E+00 | 2.56E+00 | Log-Normal |
| **PL1** | 1.18E+00 | 0.2 | 2.36E-01 | 7.79E-01 | 1.72E+00 | Log-Normal |
| **PLU1** | 6.90E-01 | 0.2 | 1.38E-01 | 4.55E-01 | 1.01E+00 | Log-Normal |
| **PF1** | 5.15E+00 | 0.3 | 1.55E+00 | 2.74E+00 | 8.87E+00 | Log-Normal |
| **PS1** | 6.90E-01 | 0.2 | 1.38E-01 | 4.55E-01 | 1.01E+00 | Log-Normal |
| **PR1** | 1.18E+00 | 0.2 | 2.36E-01 | 7.79E-01 | 1.72E+00 | Log-Normal |
| **PB1** | 5.74E+00 | 0.2 | 1.15E+00 | 3.79E+00 | 8.36E+00 | Log-Normal |
| **ALPHAL** | 2.00E-02 | 0.2 | 4.00E-03 | 1.20E-02 | 2.80E-02 | Normal |
| **ALPHALU** | 3.00E-02 | 0.2 | 6.00E-03 | 1.80E-02 | 4.20E-02 | Normal |
| **BETA** | 3.30E-01 | 0.2 | 6.60E-02 | 1.98E-01 | 4.62E-01 | Normal |
| **VMAXC** | 7.99E+00 | 0.2 | 1.60E+00 | 5.27E+00 | 1.16E+01 | Log-Normal |
| **KM** | 4.00E-02 | 0.3 | 1.20E-02 | 2.13E-02 | 6.89E-02 | Log-Normal |
| **VMAXCLU** | 1.20E-01 | 0.2 | 2.40E-02 | 7.92E-02 | 1.75E-01 | Log-Normal |
| **KMLU** | 2.10E-01 | 0.3 | 6.30E-02 | 1.12E-01 | 3.62E-01 | Log-Normal |
| **VMAXC1** | 1.26E+01 | 0.2 | 2.51E+00 | 8.29E+00 | 1.83E+01 | Log-Normal |
| **KM1** | 2.18E+00 | 0.3 | 6.54E-01 | 1.16E+00 | 3.76E+00 | Log-Normal |
| **VMAXCLU1** | 7.50E-01 | 0.2 | 1.50E-01 | 4.95E-01 | 1.09E+00 | Log-Normal |
| **KMLU1** | 5.38E+00 | 0.3 | 1.61E+00 | 2.86E+00 | 9.27E+00 | Log-Normal |
| **VMAXC1O** | 2.66E+00 | 0.2 | 5.32E-01 | 1.76E+00 | 3.88E+00 | Log-Normal |
| **KM1O** | 1.76E+00 | 0.3 | 5.28E-01 | 9.37E-01 | 3.03E+00 | Log-Normal |
| **KGSHLC** | 1.30E-01 | 0.2 | 2.60E-02 | 8.58E-02 | 1.89E-01 | Log-Normal |
| **KGSHLUC** | 1.30E-01 | 0.2 | 2.60E-02 | 8.58E-02 | 1.89E-01 | Log-Normal |
| **LLOXACT** | 4.20E-01 | 0.2 | 8.40E-02 | 2.52E-01 | 5.88E-01 | Normal |
| **LLEEACT** | 1.40E-01 | 0.2 | 2.80E-02 | 8.40E-02 | 1.96E-01 | Normal |
| **KFEEC** | 3.50E+01 | 0.2 | 7.00E+00 | 2.31E+01 | 5.10E+01 | Log-Normal |
| **KPC** | 6.00E-02 | 0.2 | 1.20E-02 | 3.96E-02 | 8.74E-02 | Log-Normal |
| **GSO** | 7.00E+03 | 0.2 | 1.40E+03 | 4.20E+03 | 9.80E+03 | Normal |
| **GSOLU** | 1.50E+03 | 0.2 | 3.00E+02 | 9.00E+02 | 2.10E+03 | Normal |

| **Table S7: Summary of the MC analysis of model uncertainty for female mouse (1000 iterations of the MC model, 6 hours/day, 5 days/week, 14 days)** | | | | | |
| --- | --- | --- | --- | --- | --- |
|  | **Mean** | **SD** | **CV%** | **median** | **95% CI** |
| **12.8 ppm** | | | | | |
| **Tmet** | 9.00E-01 | 4.17E-01 | 46% | 8.24E-01 | 3.57E-01 - 1.94E+00 |
| **Preact** | 4.96E-01 | 1.14E+00 | 230% | 2.47E-01 | 8.05E-02 - 2.48E+00 |
| **1-CEO** | 6.16E-04 | 2.38E-04 | 39% | 5.74E-04 | 2.70E-04 - 1.21E-03 |
| **32 ppm** | | | | | |
| **Tmet** | 1.36E+00 | 5.77E-01 | 43% | 1.26E+00 | 6.10E-01 - 2.79E+00 |
| **Preact** | 1.36E+00 | 2.58E+00 | 189% | 4.95E-01 | 1.44E-01 - 8.12E+00 |
| **1-CEO** | 1.29E-03 | 5.33E-04 | 41% | 1.18E-03 | 5.41E-04 - 2.70E-03 |
| **80 ppm** | | | | | |
| **Tmet** | 1.75E+00 | 7.09E-01 | 41% | 1.61E+00 | 8.17E-01 - 3.65E+00 |
| **Preact** | 2.57E+00 | 4.04E+00 | 157% | 8.83E-01 | 2.10E-01 - 1.47E+01 |
| **1-CEO** | 2.76E-03 | 1.24E-03 | 45% | 2.51E-03 | 1.07E-03 - 6.10E-03 |

Tmet: average amount chloroprene metabolized per gram lung per day (μmol/g lung/day)

Preact: average concentration reactive product in lung (μM)

1-CEO: average concentration 1-CEO in lung (μM)

**Chloroprene model code for the epoxy submodel**

#Chloroprene PBPK Model

#Translated from the acslX model presented in Yang et al. 2012

#By Jerry Campbell 2019

#Added 1-CEO, Reaction Product and GSH submodels

#By Jerry Campbell 2021

States = {

AI ,

AX ,

AM ,

AMCP_1CE ,

AMCP_2CE ,

AMLU ,

AMLUCP_1CE ,

AMLUCP_2CE ,

AMK ,

ALU ,

AL ,

AK ,

AS ,

AR ,

AUCCR ,

AF ,

AX1 ,

AM1 ,

AMLU1 ,

AM1O ,

AMLU1O ,

ALUE1 ,

ALU1 ,

ALE1 ,

AL1 ,

AS1 ,

AR1 ,

AF1 ,

ARPG ,

ARPGLU ,

ARPEE ,

ARPEELU ,

ALRPPRO ,

ALRP ,

ALURPPRO ,

ALURP ,

AGSHL ,

AGSHLU ,

AUCCLRP ,

AUCCLURP ,

AUCCEO1L ,

AUCCEO1LU ,

AUCGSHL ,

AUCGSHLU

};

Outputs = {

CVL ,

GSHL ,

GSHLU ,

CLRP ,

CLURP ,

VL ,

MASBAL ,

A1CEOGEN ,

MASBAL1 ,

CLU ,

CL ,

CK ,

CS ,

CR ,

CF ,

CV ,

CVLUM ,

CVLUM1 ,

CLUE1 ,

CLU1 ,

CLE1 ,

CL1 ,

CS1 ,

CR1 ,

CF1 ,

CV1 ,

qcbal ,

vbal ,

ppm ,

AMP ,

AMPLU ,

AMPK ,

AM1L ,

AM1LU ,

AM1LO ,

AM1LUO ,

ARPL ,

ARPLU ,

ARPOTHL ,

ARPOTHLU ,

ARPGSHL ,

ARPGSHLU ,

CLRPAVG ,

CLURPAVG ,

CL1AVG ,

CLU1AVG ,

GSHLAVG ,

GSHLUAVG ,

RALURPPRO

};

Inputs = {EXPPULSE} ;

#BODY WEIGHT (kg)

BW = 0.03 ; # Body weight (kg)

#SPECIAL FLOW RATES

QPC = 29.1 ; # Unscaled Alveolar Vent (L/h/kg^0.75)

QCC = 20.1 ; # Unscaled Cardiac Output (L/h/kg^0.75)

#FRACTIONAL BLOOD FLOWS TO TISSUES

QLC = 0.161 ; # Flow to Liver as % Cardiac Output (unitless)

QFC = 0.07 ; # Flow to Fat as % Cardiac Output (unitless)

QSC = 0.159 ; # Flow to Slow as % Cardiac Output (unitless)

QKC = 0.09 ; # Flow to Kidney as % Cardiac Output (unitless)

QRC = 0.52 ; # Flow to Rapid as % Cardiac Output (unitless)

#FRACTIONAL VOLUMES OF TISSUES

VLC = 0.055 ; # Volume Liver as % Body Weight (unitless)

VLUC = 0.0073 ; # Volume Lung as % Body Weight (unitless)

VFC = 0.1 ; # Volume Fat as % Body Weight (unitless)

VRC = 0.08098 ; # Volume Rapid Perfused as % Body Weight (unitless)

VSC = 0.384 ; # Volume Slow Perfused as % Body Weight (unitless)

VKC = 0.0167 ; # Volume Kidney as % Body Weight (unitless)

VTOT_B = 0.64398 ; # Balance for tissue MC

#PARTITION COEFFICIENTS PARENT

#Chloroprene

PL = 1.26 ; # Liver/Blood Partition Coefficient (unitless)

PLU = 2.38 ; # Lung/Blood Partition Coefficient (unitless)

PF = 17.35 ; # Fat/Blood Partition Coefficient (unitless)

PS = 0.59 ; # Slow/Blood Partition Coefficient (unitless)

PR = 1.76 ; # Rapid/Blood Partition Coefficient (unitless)

PB = 7.83 ; # Blood/Air Partition Coefficient (unitless)

PK = 1.76 ; # Kidney/Blood Partition Coefficient (unitless)

#PARTITION COEFFICIENTS 1-CEO (IndusChemFate, LogKow 1.22)

PL1 = 1.26 ; # Liver/Blood Partition Coefficient (unitless)

PLU1 = 2.38 ; # Lung/Blood Partition Coefficient (unitless)

PF1 = 17.35 ; # Fat/Blood Partition Coefficient (unitless)

PS1 = 0.59 ; # Slow/Blood Partition Coefficient (unitless)

PR1 = 1.76 ; # Rapid/Blood Partition Coefficient (unitless)

PB1 = 7.8 ; # Blood/Air Partition Coefficient (unitless)

#KINETIC CONSTANTS

MW = 88.5 ; # Molecular weight (g/mol)

MWCEO = 104.5 ; # 1-CEO Molecular weight (g/mol)

#Chloroprene

#Fraction of total metabolism to 1-CEO

ALPHAL = 0.02 ; #Fraction of liver chloroprene metabolism to 1-CEO

ALPHALU = 0.03 ; #Fraction of lung chloroprene metabolism to 1-CEO

#Fraction of total CP to 1-CEO privaleged access

#(based on butadiene model Campbell et al. 2015; assumed same in liver and lung)

BETA = 0.67 ;

# CP Metabolism in Liver

VMAXC = 7.95 ; # Scaled VMax for Oxidative Pathway:Liver (mg/h/BW^0.75)

KM = 0.041 ; # Km for Oxidative Pathway:Liver (mg/L)

# CP Metabolism in Lung

VMAXCLU = 0.18; # Scaled VMax for Oxidative Pathway:Lung (mg/h/BW^0.75)

KMLU = 0.26; # Km for Oxidative Pathway:Lung (mg/L)

# CP Metabolism in Kidney

VMAXCKid = 0.0 ; # Scaled VMax for Oxidative Pathway:Kidney (mg/h/BW^0.75)

KMKD = 1.0 ; # Km for Oxidative Pathway :Kidney

#1-CEO

#1-CEO Metabolism in Liver - Hydrolysis

VMAXC1 = 7.95 ; # Scaled VMax for Hydrolysis Pathway:Liver (mg/h/BW^0.75)

KM1 = 0.041 ; # Km for Hydrolysis Pathway:Liver (mg/L)

#1-CEO Metabolism in Lung - Hydrolysis

VMAXCLU1 = 0.18; # Scaled VMax for Hydrolysis Pathway:Lung (mg/h/BW^0.75)

KMLU1 = 0.26; # Km for Hydrolysis Pathway:Lung (mg/L)

#1-CEO Metabolism in Liver - Oxidative (Mouse Only!!!)

VMAXC1O = 7.95 ; # Scaled VMax for Oxidative Pathway:Liver (mg/h/BW^0.75)

KM1O = 0.041 ; # Km for Oxidative Pathway:Liver (mg/L)

#Reactive Products

#Liver

KGSHLC = 0.0 ; #2nd order rate of RP reaction with GSH

K3L = 0.0 ; #Reaction rate with cellular macromolecules

MML = 0.0 ; #macromolecule concentration (mM)

#Lung

KGSHLUC = 0.0 ; #2nd order rate of RP reaction with GSH (L/mmol/hr)

K3LU = 0.0 ; #Reaction rate with cellular macromolecules (L/mmol/hr)

MMLU = 0.0 ; #macromolecule concentration (mM)

#GSH

K0L = 0.0 ; #Production of GSH

K1L = 0.0 ; #Background loss of GSH

K0LU = 0.0 ; #Production of GSH

K1LU = 0.0 ; #Background loss of GSH

#Permeation Coefficient (fraction of blood flow)

PA1 = 1.0 ; #Permeation Coefficient for 1-CEO in lung

#DOSING INFORMATION

TSTOP = 7.0 ; # Dosing stop time

CONC = 13.0 ; # Initial concentration (ppm)

#Parameters for GSH submodel

LLOXACT = 0.14 ; #Scaler for liver to lung oxidative metabolism 1-CEO (mouse only)

LLEEACT = 0.06 ; #Scaler for liver to lung oxidative metabolism 1-CEO (mouse only)

KFEEC = 4500.0 ; # 1/hr/kg Conjugation rate with non-GSH

KPC = 0.06 ; # First-order rate constant for GSH loss

GSO = 5500.0 ; # Initial GSH concentration liver

GSOLU = 1200.0 ; # Initial GSH concentration lung

Dynamics {

##################################################################################

# Scaled parameters

QP = QPC*pow(BW,0.75) ; #Alveolar ventilation

QTOT = QRC + QLC + QKC + QFC + QSC ;

QCi = QCC*pow(BW,0.75) ; #Cardiac output

QL = QLC*QCi/QTOT ; #Liver blood flow

QF = QFC*QCi/QTOT ; #Fat blood flow

QS = QSC*QCi/QTOT ; #Slowly-perf tissue blood flow

QK = QKC*QCi/QTOT ; #Kidney tissue blood flow

QR = QRC*QCi/QTOT ; #Rapily-perf tissue blood flow

QC = QL + QF + QS + QK + QR ;

VTOT = VLC + VLUC + VFC + VSC + VRC + VKC ;

VL = VLC*BW*(VTOT_B/VTOT) ; #Liver volume

VLU = VLUC*BW*(VTOT_B/VTOT) ; #Lung volume

VF = VFC*BW*(VTOT_B/VTOT) ; #Fat tissue volume

VS = VSC*BW*(VTOT_B/VTOT) ; #Slowly-perfused tissue volume

VR = VRC*BW*(VTOT_B/VTOT) ; #Richly-perfised tissue volume

VK = VKC*BW*(VTOT_B/VTOT) ; #kidney tissue volume

# METABOLISM

VMAX = VMAXC*pow(BW,0.75) ; #Maximum rate of metabolism-Liver (mg/hr)

VMAXLU = VMAXCLU*pow(BW,0.75) ; #Maximum rate of metabolism-Lung (mg/hr)

VMAXKD = VMAXCKid*pow(BW,0.75) ; #Maximum rate of metabolism-Kidney (mg/hr)

VMAX1 = VMAXC1*pow(BW,0.75) ; #Maximum rate of metabolism-Liver (mg/hr)

VMAXLU1 = VMAXCLU1*pow(BW,0.75) ; #Maximum rate of metabolism-Lung (mg/hr)

VMAX1O = VMAXC1O*pow(BW,0.75) ; #Maximum rate of metabolism-Liver (mg/hr)

VMAXLU1O = VMAX1O*LLOXACT ; #Liver Vmax scaled to lung (ECD model)

KGSHL = KGSHLC;

KFEE = KFEEC ;

KGSHLU = KGSHLUC;

KFEELU = KFEE*LLEEACT ;

KP = KPC*pow(BW,-0.3) ;

KOTDL = KP*GSO ;

KOLUTDL = KP*GSOLU ;

##################################################################################

# Exposure Control (mg/L)

CIX = CONC*MW/24450 ;

CI = CIX *EXPPULSE ;

##################################################################################

# Chloroprene

# Tissue Venous Concentrations (mg/L)

CVLU = ALU/(VLU*PLU) ;

CVL = AL/(VL*PL) ;

CVK = AK/(VK*PK) ;

CVS = AS/(VS*PS) ;

CVR = AR/(VR*PR) ;

CVF = AF/(VF*PF) ;

#Tissue Concentration (mg/L)

# 1-CEO

# Tissue Venous Concentrations (mmol/L)

CLUE1 = ALUE1/VLU ;

CVLU1 = ALU1/(VLU*PLU1) ;

CLU1 = (ALUE1+ALU1)/VLU ;

CLE1 = ALE1/VL ;

CVL1 = AL1/(VL*PL1) ;

CL1 = (ALE1 + AL1)/VL ;

CVS1 = AS1/(VS*PS1) ;

CVR1 = AR1/(VR*PR1) ;

CVF1 = AF1/(VF*PF1) ;

#Concentration of GSH in Liver and Lung

GSHL = AGSHL/VL ; #GSH concentraiton in liver (uM)

GSHLU = AGSHLU/VLU ; #GSH concentraiton in lung (uM)

##################################################################################

# Concentration in Pulmonary/Arterial and venous blood Compartments (mg/L)

CPU = (QP*CI+(QF*CVF + QL*CVL + QS*CVS + QR*CVR + QK*CVK))/(QP/PB+QC) ;

CX = CPU/PB ;

CV = (QF*CVF + QL*CVL + QS*CVS + QR*CVR + QK*CVK)/QC ;

CPUM = CPU*1000/MW ;

RAI = QP*CI ;

dt(AI) = RAI ;

RAX = QP*CX ;

dt(AX) = RAX ;

##################################################################################

##################################################################################

#Rate amount metabolized in liver, lung and kidney

# Amount metabolized in Liver (mg)

RAM = VMAX*CVL/(KM+CVL) ;

dt(AM) = RAM ;

#Total 1 and 2-CEO from CP

RAMCP_1CEO = RAM*ALPHAL ; #CP metabolized to 1-CEO in Liver

dt(AMCP_1CE) = RAMCP_1CEO ;

RAMCP_2CEO = RAM*(1-ALPHAL) ; #CP metabolized to 2-CEO in Liver

dt(AMCP_2CE) = RAMCP_2CEO ;

# Amount metabolized in Lung (mg)

RAMLU = VMAXLU*CVLU/(KMLU+CVLU) ;

dt(AMLU) = RAMLU ;

RAMLUCP_1CEO = RAMLU*ALPHALU ; #CP metabolized to 1-CEO in Lung

dt(AMLUCP_1CE) = RAMLUCP_1CEO ;

RAMLUCP_2CEO = RAMLU*(1-ALPHALU) ; #CP metabolized to 2-CEO in Lung

dt(AMLUCP_2CE) = RAMLUCP_2CEO ;

# Amount metabolized in Kidney (mg)

RAMK = VMAXKD*CVK/(KMKD + CVK) ;

dt(AMK) = RAMK ;

##################################################################################

##################################################################################

# Amount in Lung Compartment (mg)

RALU = QC*(CPU-CVLU) - RAMLU ;

dt(ALU) = RALU ;

# Amount in Liver Compartment (mg)

RAL = QL*(CVLU-CVL) - RAM ;

dt(AL) = RAL ;

# Amount in Kidney Compartment (mg)

RAK = QK*(CVLU-CVK) - RAMK ;

dt(AK) = RAK ;

# Amount in Slowly Perfused Tissues (mg)

RAS = QS*(CVLU-CVS) ;

dt(AS) = RAS ;

# Amount in Rapidly Perfused Tissues (mg)

RAR = QR*(CVLU-CVR) ;

dt(AR) = RAR ;

dt(AUCCR) = AR/VR ;

# Amount in Fat Compartment (mg)

RAF = QF*(CVLU-CVF) ;

dt(AF) = RAF ;

#########################################################################

#########################################################################

#1-CEO submodel

##################################################################################

##################################################################################

##################################################################################

# Concentration 1-CEO in Pulmonary/Arterial and venous blood Compartments (mg/L)

CV1 = (QF*CVF1 + QL*CVL1 + QS*CVS1 + (QR+QK)*CVR1)/QC ;

CPU1 = (QC*CV1)/(QP/PB1+QC) ;

CX1 = CPU1/PB1 ;

RAX1 = QP*CX1 ;

dt(AX1) = RAX1 ;

#Rate amount 1-CEO metabolized in liver and lung

#Hydrolysis (1-CEO to diol)

# Amount metabolized in Liver (mg)

RAM1 = VMAX1*CVL1/(KM1 + CVL1) ;

dt(AM1) = RAM1 ;

# Amount metabolized in Lung (mg)

RAMLU1 = VMAXLU1*CVLU1/(KMLU1 + CVLU1) ;

dt(AMLU1) = RAMLU1 ;

#Oxidative (Mouse Only!!!)

# Amount metabolized in Liver (mg)

RAM1O = VMAX1O*CVL1/(KM1O + CVL1) ;

dt(AM1O) = RAM1O ;

# Amount metabolized in Lung (mg)

RAMLU1O = VMAXLU1O*CVLU1/(KM1O + CVLU1) ;

dt(AMLU1O) = RAMLU1O ;

##################################################################################

##################################################################################

# 1-CEO in Lung Compartment

# Amount in Lung Epithelium (mmol)

RALUE1 = PA1*QC*(CVLU1 - CLUE1/PLU1) + RAMLUCP_1CEO*BETA*MWCEO/MW - RAMLU1 - RAMLU1O ;

dt(ALUE1) = RALUE1 ;

# Amount in Lung Submucosa (mmol)

RALU1 = QC*(CPU1-CVLU1) + PA1*QC*(CLUE1/PLU1 - CVLU1) ;

dt(ALU1) = RALU1 ;

# 1-CEO in Liver

# Amount in Liver Epithelium (mmol)

RALE1 = PA1*QL*(CVL1 - CLE1/PL1) + RAMCP_1CEO*BETA*MWCEO/MW - RAM1 - RAM1O ;

dt(ALE1) = RALE1 ;

# Amount in Liver Compartment (mmol)

RAL1 = QL*(CVLU1-CVL1) + PA1*QL*(CLE1/PL1 - CVL1) ;

dt(AL1) = RAL1 ;

# Amount in Slowly Perfused Tissues (mmol)

RAS1 = QS*(CVLU1 - CVS1) ;

dt(AS1) = RAS1 ;

# Amount in Rapidly Perfused Tissues (mmol)

RAR1 =(QR+QK)*(CVLU1 - CVR1) ;

dt(AR1) = RAR1 ;

# Amount in Fat Compartment (mmol)

RAF1 = QF*(CVLU1 - CVF1) ;

dt(AF1) = RAF1 ;

#########################################################################

#########################################################################

#########################################################################

#Reactive Products (converted to umol or umol/L for GSH submodel)

##################################################################################

##################################################################################

CLRP = ALRP/VL ; #(umol/L)

CLURP = ALURP/VLU ; #(umol/L)

# ACMG = AMOUNT METABOLITE CONJUGATED WITH GLUTATHIONE (UMOLES)

RARPG = KGSHL*GSHL*CLRP*VL ;

dt(ARPG) = RARPG ;

RARPGLU = KGSHLU*GSHLU*CLURP*VLU ;

dt(ARPGLU) = RARPGLU ;

# ACMEE = AMOUNT METABOLITE CONJUGATED WITH OTHER THINGS (UMOLES)

RARPEE = KFEE*CLRP*VL ;

dt(ARPEE) = RARPEE ;

RARPEELU = KFEELU*CLURP*VLU ;

dt(ARPEELU) = RARPEELU ;

#Reactive products in liver (umol)

dt(ALRPPRO) = (RAMCP_2CEO/MW)*1000 + (RAM1O/MWCEO)*1000 ;

RALRP = (RAMCP_2CEO/MW)*1000 + (RAM1O/MWCEO)*1000 - RARPG - RARPEE ;

dt(ALRP) = RALRP ;

#Reactive products in lung (umol)

RALURPPRO = (RAMLUCP_2CEO/MW)*1000 + (RAMLU1O/MWCEO)*1000 ;

dt(ALURPPRO) = RALURPPRO ;

RALURP = (RAMLUCP_2CEO/MW)*1000 + (RAMLU1O/MWCEO)*1000 - RARPGLU - RARPEELU ;

dt(ALURP) = RALURP ;

#########################################################################

#########################################################################

#GSH

##################################################################################

##################################################################################

#GSH in liver (umol)

RAGSHL = KOTDL*VL - KP*GSHL*VL - RARPG ;

dt(AGSHL) = RAGSHL ;

#GSH in LU (umol)

RAGSHLU = KOLUTDL*VLU - KP*GSHLU*VLU - RARPGLU ;

dt(AGSHLU) = RAGSHLU ;

#AUCs for reactive products and 1-CEO:

dt(AUCCLRP) = CLRP ; #uM*hr

dt(AUCCLURP) = CLURP ; #uM*hr

dt(AUCCEO1L) = CL1/MWCEO*1000 ; #uM*hr

dt(AUCCEO1LU) = CLU1/MWCEO*1000 ; #uM*hr

dt(AUCGSHL) = GSHL ; #uM*hr

dt(AUCGSHLU) = GSHLU ; #uM*hr

##################################################################################

##################################################################################

##################################################################################

} # End of Dynamics

#########################################################################

#########################################################################

#########################################################################

CalcOutputs {

# Mass-balance

MASBAL = AI - AX - (AL+AM+AMLU+AK+AMK+AS+AR+AF+ALU) ;

A1CEOGEN = AM*ALPHAL*(1-BETA)*MWCEO/MW + AMLU*ALPHALU*(1-BETA)*MWCEO/MW ;

MASBAL1 = A1CEOGEN - AX1 - (AM1+AMLU1+AM1O+AL1+AS1+AR1+AF1+ALU1) ;

#Tissue Concentrations (mg/L)

CLU = ALU/VLU ;

CL = AL/VL ;

CK = AK/VK ;

CS = AS/VS ;

CR = AR/VR ;

CF = AF/VF ;

#Concentrations for plots

CVLUM = CV*1000/MW ; #(umol/L)

CVLUM1 = CV1*1000/MWCEO ; #(umol/L)

#Tissue Concentrations 1-CEO (mg/L)

CS1 = AS1/VS ;

CR1 = AR1/VR ;

CF1 = AF1/VF ;

#Blood Flow balance

qcbal = QC - QL - QF - QS - QK - QR ;

#Tissue Volume balance

vbal = BW*VTOT_B - VL - VLU - VF - VS - VK - VR ;

#Dose metrics are only correct when simulation time=tstop

ppm = CONC ;

#Total Metabolism umol/g/day

AMP = ((AM*1000/MW)/(VL*1000))/(TSTOP/24) ;

AMPLU = ((AMLU*1000/MW)/(VLU*1000))/(TSTOP/24) ;

AMPK = ((AMK*1000/MW)/(VK*1000))/(TSTOP/24) ;

#Hydrolase Metabolism of 1-CEO (umol/g/day)

AM1L = ((AM1*1000/MWCEO)/(VL*1000))/(TSTOP/24) ;

AM1LU = ((AMLU1*1000/MWCEO)/(VLU*1000))/(TSTOP/24) ;

#Oxidative metabolims of 1-CEO umol/g/day

AM1LO = ((AM1O*1000/MWCEO)/(VL*1000))/(TSTOP/24) ;

AM1LUO = ((AMLU1O*1000/MWCEO)/(VLU*1000))/(TSTOP/24) ;

#Total production of RP (umol/g/day)

ARPL = ((ALRPPRO)/(VL*1000))/(TSTOP/24) ;

ARPLU = ((ALURPPRO)/(VLU*1000))/(TSTOP/24) ;

#Total reaction of RP with other (umol/g/day)

ARPOTHL = ((ARPEE)/(VL*1000))/(TSTOP/24) ;

ARPOTHLU = ((ARPEELU)/(VLU*1000))/(TSTOP/24) ;

#Total reaction of RP with GSH (umol/g/day)

ARPGSHL = ((ARPG)/(VL*1000))/(TSTOP/24) ;

ARPGSHLU = ((ARPGLU)/(VLU*1000))/(TSTOP/24) ;

#Average concentration of RP (uM)

CLRPAVG = AUCCLRP/TSTOP ;

CLURPAVG = AUCCLURP/TSTOP ;

#Average concentration of 1-CEO (uM)

CL1AVG = AUCCEO1L/TSTOP ;

CLU1AVG = AUCCEO1LU/TSTOP ;

#Average concentration of GSH (uM)

GSHLAVG = AUCGSHL/TSTOP ;

GSHLUAVG = AUCGSHLU/TSTOP ;

} # End of CalcOutputs

End.
